# Dissecting the genetic basis of bioactive metabolites and fruit quality traits in blueberries (*Vaccinium corymbosum* L.)

**DOI:** 10.3389/fpls.2022.964656

**Published:** 2022-09-02

**Authors:** Molla Fentie Mengist, Mary H. Grace, Ted Mackey, Bryan Munoz, Boas Pucker, Nahla Bassil, Claire Luby, Mario Ferruzzi, Mary Ann Lila, Massimo Iorizzo

**Affiliations:** ^1^Plants for Human Health Institute, North Carolina State University, Kannapolis, NC, United States; ^2^Department of Food, Bioprocessing and Nutrition Sciences, North Carolina State University, Raleigh, NC, United States; ^3^Horticultural Crops Research Unit, U.S. Department of Agriculture, Agricultural Research Service, Corvallis, OR, United States; ^4^Institute of Plant Biology, TU Braunschweig, Braunschweig, Germany; ^5^BRICS, TU Braunschweig, Braunschweig, Germany; ^6^National Clonal Germplasm Repository, USDA-ARS, Corvallis, OR, United States; ^7^Department of Horticultural Science, North Carolina State University, Raleigh, NC, United States

**Keywords:** blueberry, anthocyanin, acylation, glycosylation, QTL mapping, candidate genes, fruit quality, chlorogenic acid

## Abstract

Blueberry is well-recognized as a healthy fruit with functionality derived largely from anthocyanin and chlorogenic acid. Despite their importance, no study to date has evaluated the genetic basis of these bioactives in blueberries and their relationship with fruit quality traits. Hence, to fill this gap, a mapping population including 196 F_1_ individuals was phenotyped for anthocyanin and chlorogenic acid concentration and fruit quality traits (titratable acidity, pH, and total soluble solids) over 3 years and data were used for QTL mapping and correlation analysis. Total soluble solids and chlorogenic acid were positively correlated with glycosylated anthocyanin and total anthocyanin, respectively, indicating that parallel selection for these traits is possible. Across all the traits, a total of 188 QTLs were identified on chromosomes 1, 2, 4, 8, 9, 11 and 12. Notably, four major regions with overlapping major-effect QTLs were identified on chromosomes 1, 2, 4 and 8, and were responsible for acylation and glycosylation of anthocyanins in a substrate and sugar donor specific manner. Through comparative transcriptome analysis, multiple candidate genes were identified for these QTLs, including glucosyltransferases and acyltransferases. Overall, the study provides the first insights into the genetic basis controlling anthocyanins accumulation and composition, chlorogenic acid and fruit quality traits, and establishes a framework to advance genetic studies and molecular breeding for anthocyanins in blueberry.

## Introduction

Blueberry is well-recognized as a health protective fruit with functionality derived largely from bioactives, in particular anthocyanin and chlorogenic acid (CHA; Grace et al., 2019; Kalt et al., 2020). These multiple health benefits made blueberry very popular among consumers which likely contributed to the rapid increase in consumption and production in the US and globally during the last 10 years (Fulcher et al., 2015; Mengist et al., 2021). Furthermore, blueberries are consumed in both fresh and processed forms (e.g., frozen, dried, preserves) allowing integration in consumer diets for highest potential impact. Given their importance, multiple studies have evaluated the anthocyanin concentration and composition and CHA concentration in blueberry and their nutrigenomic properties (Grace et al., 2019; Mengist et al., 2020a, 2020b) opening opportunities and rationale to breed to these traits in blueberry. Breeding for anthocyanin concentration and profile as well as other bioactives like CHA depends on the availability of genetic variation for the trait within the germplasm. The anthocyanin profile in varieties of highbush blueberry contains around 18–20 types of anthocyanins (Grace et al., 2019; Mengist et al., 2020a,b). Five aglycones including Delphinidin (Dp), Cyanidin (Cyn), Petunidin (Pet), Peonidin (Peo) and Malvidin (Mv), and three sugar moieties namely arabinose (arab), galactose (gal) and glucose (glu) are commonly found in blueberries (Grace et al., 2019; Mengist et al., 2020b). Acylation of anthocyanins also adds another layer of anthocyanin structural diversity (Grace et al., 2019; Mengist et al., 2020a,b) in blueberry. Previous studies demonstrated that extensive phenotypic variation for anthocyanin concentrations and composition, and CHA concentration exist in blueberry germplasm (Yousef et al., 2016; Grace et al., 2019; Mengist et al., 2020a,b). Our previous studies demonstrated that acylation and glycosylation are important variables for clustering accessions into different groups, and CHA concentration, anthocyanin concentration and profiles had a moderate to high heritability (Mengist et al., 2020a,b). Our recent study revealed that acylated anthocyanins have higher bioaccessibility than non-acylated anthocyanins (Mengist et al., 2020a), suggesting that consumption of blueberry with high acylated anthocyanin could increase absorption of these bioactives. Furthermore, acylated anthocyanins have higher color stability than the counterpart non-acylated anthocyanins, which is particularly relevant for the application of anthocyanin as a natural colorant in the food industries (Matera et al., 2015; Zhao et al., 2017; Strauch et al., 2019).

Quantitative trait loci (QTL) mapping is a commonly used method to identify the genetic basis of important agronomic, nutritional and stress resistance traits (Bourke et al., 2018; Mengist et al., 2021). However, efforts to perform QTL mapping in autotetraploid species like blueberry have been limited due to the complexity of their genome and chromosome segregation pattern, which limited the use of tools for linkage map and QTL analysis that were largely developed for diploid organisms (Bourke et al., 2018). Recent development of genotyping platform such as capture-seq, high-quality genome assembly and statistical tools to develop linkage maps and QTL mapping in autopolyploid species provided opportunities to develop high-density genetic maps and advance QTL mapping in blueberry (Bourke et al., 2018; Mengist et al., 2021). For instance, a recent study from Mengist et al., described a high density linkage map for blueberry and the first QTL mapping in tetraploid blueberry for fruit quality traits (pH, TA, TSS). However, the genetic mechanisms controlling CHA concentration, anthocyanin concentration and profile (acylation and glycosylation) in blueberries are still unknown. As a consequence it is unknown how the genetic mechanisms controlling anthocyanin accumulation interact with those controlling fruit quality traits like pH, TA, TSS.

Although the genetic mechanism controlling anthocyanin concentration and composition, and CHA concentration in blueberry has not been studied to date, multiple studies annotated and characterized genes involved in anthocyanin regulation and biosynthesis. Thirty-five transcription factors (TF) and over 90 genes associated with anthocyanin and CHA biosynthesis and transport were annotated in the blueberry genome and were characterized by gene expression analysis (Colle et al., 2019). A MYB transcription factor has been functionally characterized (Plunkett et al., 2018; Lafferty et al., 2022). However none of these studies evaluated if any of these genes are allelic in the blueberry germplasm and underlie any QTLs for anthocyanin and CHA concentration. Also, efforts to study genes involved in anthocyanin modification, including acylation and glycosylation are limited. Numerous studies in plants reported that glycosylation and acylation are synthetized by the activity of structural genes, and more specifically by so called Late Biosynthesis Genes (LBGs) (Jaakola, 2002, 2013; Zifkin et al., 2012). The LBGs coding enzymes such as glycosyltransferases and acyltransferases, catalyze the addition of sugar moieties and acyl groups, respectively (Iorizzo et al., 2020). UDP glucose-flavonoid 3-O-glucosyl transferase (UFGT) enzymes are one of the glycosyltransferase enzymes that are required for glycosylation of anthocyanins in plants (Jaakola, 2002, 2013; Morita et al., 2005; Zifkin et al., 2012; Cheng et al., 2014; Iorizzo et al., 2020). To date one study identified possible functional flavonoid 3-O-glucosyltransferase (*VcUFGT*) enzyme involved in anthocyanin glycosylation in blueberries (Zifkin et al., 2012). However it is unknown if the gene regulates phenotypic variation observed for anthocyanin composition.

Hence, the objectives of this study were to investigate the genetic basis of differences in anthocyanin concentration and composition, CHA concentration and fruit quality (FQ) traits, and establish the genetic association between these traits. Gene expression analysis was also performed to identify candidate genes underlying QTLs associated with anthocyanin acylation and glycosylation. QTL analysis was performed using a mapping population called DSxJ, derived from a cross between two blueberry cultivars, Draper-selection-44392 (DS) and Jewel (J). The DSxJ mapping population and parents were genotyped using 29 k SNP markers and evaluated for anthocyanins, CHA and FQ traits over 3 years. In addition, the QTL mapping study was integrated with RNA sequencing (RNA-seq) to identify potential candidate genes associated with anthocyanin composition.

## Materials and methods

### Plant materials

The mapping population is an F_1_ developed by crossing the two parents, Draper-selection-44392 (DS) and Jewel (J). The population included 196 F_1_ seedlings that were grown in Corvallis, Oregon, United States. The parent DS is a northern highbush blueberry genotype selected from Draper an early to mid-season northern highbush cultivar producing high yields of premium quality, firm and sweet fruit with superior shelf-life. Jewel is an early to mid-season southern highbush cultivar with a high yielding plant producing very large, and slightly tart fruit (Hancock et al., 2018). A previous study by (Hancock et al., 2018) indicated that the population was segregating for anthocyanin concentration, fruit quality traits (e.g., pH, TA, TSS) and physiological traits (e.g., chilling requirement). Based on this information preliminary analysis of a subset of the population (N = 60) and the two parents was performed to further evaluate segregation for anthocyanin and CHA concentration and profile. The results revealed that the two parents differed for anthocyanin concentration and composition, and CHA concentration (Supplementary Figure S1). The F_1_ genotypes also segregated for anthocyanin concentration and composition, and CHA concentration (Supplementary Figure S1). Based on this preliminary information, for this study the full set of plants available for this mapping population (N = 196) was evaluated for three consecutive years (2017–2019). Berries from these F_1_ progeny were harvested when fully ripe as indicated by the surface of the skin of the berries being completely blue. Then, the berries were stored at −80°C until processing. Frozen berries (three replicates of approximately 10–30 g each), were used to evaluate anthocyanins, CHA and FQ traits including pH, TSS and TA.

### FQ trait phenotyping

FQ traits including TSS, pH and TA were evaluated with the same procedures as described in (Mengist et al., 2020a,b, 2021). Briefly, the berries were homogenized to a puree in a Waring Commercial Blender 7012 G (Torrington, CT, United States). Then, we used the homogenized samples to estimate TSS, TA, and pH. TSS (expressed as °Brix) was measured using a digital hand-held ‘pocket’ refractometer PAL-1 (Atago, Tokyo, Japan). For pH and TA, 1 g of homogenized sample was diluted with 30 ml pre-boiled double distilled water. An Accumet AB15, pH-meter (Fisher Scientific, Waltham, MA, United States) and a Mettler DL15 Auto-Titrator (Columbus, OH, USA) were used to estimate pH and TA, respectively. TA was estimated at a pH of 8.2 using 0.02 mol L–1 sodium hydroxide and milliequivalent factor value 0.064. The TA was expressed as the percentage of citric acid (wt/wt) per 1 g FW.

### Extraction and quantification of anthocyanins and CHA

An aliquot (3 g) of the homogenized blueberry puree was weighed in a 30-mL centrifuge tube. After the addition of 8 ml of 80% methanol in water (containing 5% formic acid), this mix was homogenized using a PRO0250 (PRO Scientific Inc., Oxford, CT, United States) for 2 min to extract polyphenols. The homogenate was centrifuged (Sorvall RC-6 plus, Asheville, NC, United States) for 2 min at 4,000 rpm. The supernatant was collected in a 25-ml volumetric flask. The residue was then extracted two more times, once with 8 ml of the same solvent, and then with 100% methanol. Supernatants were collected and brought to a final volume of 25 ml. About 1 ml of each sample was diluted with equal volume of methanol–water-formic acid, 65:35:5 and filtered (0.22 μm PTFE membrane) prior to HPLC-PDA analysis for anthocyanins and chlorogenic acid.

HPLC analysis was conducted to quantify anthocyanins and chlorogenic acid. Standards, cyanidin-3-galactoside, cyanidin-3-glucoside, and malvidin-3-galactoside, were obtained from Chromadex (Irvine, CA, United States). Delphinidin-3-glucoside was purchased from Cayman Chemicals (Ann Arbor, MI, United States). Delphinidin-3-galactoside, malvidin-3-glucoside, petunidin-3-glucoside, myricetin-3-glucoside, kaempferol-3-glucoside, and syringetin-3-glucoside were obtained from Extrasynthese (Genay Cedex, France). Cyanidin-3-arabinoside and peonidin-3-glucoside were obtained from Polyphenols (Sandnes, Norway).

Each of the nine anthocyanin reference compounds and chlorogenic acid standard were individually dissolved in methanol–water-formic acid, 65:35:5, at a concentration of 5 mg/ml. Equal volumes from each standard solution were mixed together and diluted with the solvent mix to prepare a standard stock mix solution (200 μg/ml). Eight standard working solutions, used for the calibration curve, were prepared by appropriate dilution of the stock mix solution (2–175 μg/ml). The reference standard mix dilutions were injected to generate an eight-point calibration curve for each compound, separately. Standard curves were linear with R^2^ > 0.9997 ± 0.0007.

The chromatography was conducted on an Agilent 1260 HPLC with diode array detector (DAD) (Agilent Technologies, Santa Clara, CA, United States). Separation of anthocyanins was performed on a Supelco C-18 column (25 cm × 4.6 mm × 5 μm), and the temperature of the column oven was maintained at 30°C. The eluents were water (formic acid 5%, v/v) (A) and methanol (B), with a gradient of 10–20% B (0–5 min), 20–25% B (5–20 min), 25–30% B (20–25 min), 30–35% B (25–30 min), 35–90% (30–43 min), and isocratic at 90% B (43–46 min). The column was then re-equilibrated for 4 min at 5% B, at the flow rate of 1 ml/min. Absorption was recorded at 520 nm for anthocyanins, and 280 nm for chlorogenic acid. Not all anthocyanins present in blueberry are commercially available; therefore, anthocyanins with no standard reference were quantified as their corresponding glucoside or galactoside equivalent. The lowest limit of detection (LLD) was in the range of 1.24-1.91 ppm for anthocyanins, and 0.96 ppm for chlorogenic acid.

### Anthocyanins, CHA and FQ data analysis

To evaluate the degree of phenotypic variation between F_1_ genotypes, we computed a minimum, maximum, mean and fold changes of variation for all anthocyanins, CHA and FQ traits. Genotype means over years were estimated using best linear unbiased estimate (BLUE), with both genotype and year considered as fixed factors. Broad-sense heritability was estimated using variance components calculated from the restricted maximum likelihood (REML), calculated as follows:


$$H^{2}=\frac{\partial_{g}^{2}}{\partial_{g}^{2}+\frac{\partial_{gy}^{2}}{y}+\frac{\partial_{e}^{2}}{ry}}$$

where *δg*^2^, *δe*^2^, and *δgy*^2^ are variance components of genotypes, plot-to-plot variation of residuals and [genotype × environment] interaction, respectively; y is the number of environments (number of years in this study, =3) and r is the number of replications (=3). The relationship between traits was calculated using the Pearson Coefficient of Correlation using BLUE and three-year data, independently. The correlation was visualized using the R package corrplot (Wei et al., 2017). Hierarchical clustering (HC) was performed with the Spearman and Ward’s methods, and were visualized as a heatmap with a dendrogram using the heatmap.2 R package (Warnes et al., 2016).

### QTL mapping

QTL mapping was performed using a linkage map described by (Mengist et al., 2022) that includes 29 K markers mined from capture-seq method. Linkage map information included SNP dosage information and the phases of the eight parental haplotypes.

The QTL mapping was performed using R package polyqtlR (Bourke et al., 2021). This package performs QTL interval mapping in F_1_ mapping population of outcrossing autopolyploids using identity by descent probabilities. Significance thresholds of LOD values were determined using a genome-wide permutation test with 1,000 permutations (*α* = 0.05). As significant QTLs were detected, the initial significant QTL was included as cofactor and the QTL analysis was re-run to check if additional QTLs could be detected in other positions. The inclusion of significant QTLs as cofactors helps to reduce background variance in the phenotypic data, which can increase the power to detect QTLs in the other positions. Once the significant QTLs were confirmed, the QTL was further explored to determine the most likely QTL configuration (the parental origin of QTL alleles that have an effect on the phenotype), phenotypic variance explained by the QTL and direction of QTL effect (positive or negative; Bourke et al., 2018; Mengist et al., 2018). The best simple model was identified using Bayesian Information Criterion (BIC). The BIC gives a measure of the likelihood of the different QTL models tested, and the best simple model is considered the one with the lowest BIC value, as described by (Bourke et al., 2018; Mengist et al., 2021).

### RNA-seq analysis and candidate gene analysis

For RNA-seq study, eight F_1_ clones were selected from DSxJ mapping population based on 3 years anthocyanin data. Samples were selected to represent contrasting profiles for the anthocyanin acylation and glycosylation. More specifically for glycosylation, four genotypes (DxJ_104, DxJ_149, DxJ_49 and DxJ_160) were used to represent the low-glucoside based anthocyanin. Three genotypes (DxJ_90, DxJ_137 and DxJ_232) were used to represent the high-glucoside based anthocyanin. These two sets of samples are also representing low vs. high galactoside (Cy-Peo-Mv). Transcriptome comparison between these two sets of samples was used to identify candidate genes underlying the QTLs mapped on chromosome 1, 4 and 8, controlling anthocyanin glycosylation. Regarding acylation, three samples (DxJ_90, DxJ_137 and DxJ_232) were used to represent the high-acylated group while four clones (DxJ_104, DxJ_140, DxJ_149, and DxJ_160) were used to represent the low-acylated group. Transcriptome comparison between these two sets of samples was used to identify candidate genes underlying the QTL mapped on chromosome 2 controlling anthocyanin acylation.

Total RNA was extracted from fruit of eight genotypes using the Sigma-Aldrich kit (Sigma-Aldrich, Missouri, United States) following the manufacturer’s protocol. Library preparation and RNA-sequencing were performed by Novogene (Novogene, CA, USA). Briefly, RNA library was prepared by polyA capture (or rRNA removal) and reverse transcription of cDNA. RNA-sequencing was performed strand-specific and a150 bp paired-end sequencing strategy using an Illumina NovaSeq 6000. The RNA-seq reads were trimmed with Trimmomatic version 0.39 (Bolger et al., 2014) with default setting. The cleaned RNA-seq data were aligned to the W85_v2 reference genome sequence (Mengist et al., 2022) using STAR-2.7.10a (Dobin et al., 2012) and expression levels were quantified using StringTie V1.3.6 (Pertea et al., 2016). DESeq2 was used for differential analysis. DEGs were identified if ‘*P*-adjust <0.05 and|log2 Fold Change| >1’ (Love et al., 2014). Functional annotation of DEGs was performed using eggNOG-mapper (Huerta-Cepas et al., 2017), which is a tool that predicts gene function based on fast orthology assignments.

In order to narrow-down the list of candidate genes associated with the two conditions, glycosylation and acylation, the QTL results were integrated with the RNA-seq data. We set criteria to determine whether the genes are potential candidate genes or not. For this, the genomic regions were delimited based on two-LOD support intervals (narrow region close to the peak, and considered as first priority gene list), and genes located within the 95% permutation support interval (considered as second priority genes). To search for candidate genes, genes involved in anthocyanin biosynthesis and decoration were annotated. Annotation of genes involved in anthocyanin biosynthesis was performed by Knowledge-based Identification of Pathway Enzymes (KIPEs) v0.34 (Pucker et al., 2020). Anthocyanin biosynthesis regulating MYB transcription factors (TFs) were annotated using MYB_annotator v0.205 (Pucker, 2022). The annotation of bHLH and TTG1 TFs was carried with collect_best_BLAST_hits.py (Pucker et al., 2022) followed by the construction of an alignment with MAFFT (Katoh and Standley, 2013) and a phylogenetic tree with FastTree2 (Price et al., 2010). The bait sequences for the identification are a customized collection of bHLH land mark sequences. For genes involved in the decoration of the anthocyanins including glycosylation and acylation, the sequence of genes (peptide sequences) located within the region spanning the QTL intervals were extracted and aligned against a local database including putative members of the UFGT, SCP/SCPL and BAHD gene families from numerous plant species. Genes that had >80% similarity with UFGTs and acyltransferase genes were extracted and used for phylogenetic analysis. Phylogenetic analysis was conducted using MEGA version 7.0.26 (Kumar et al., 2016) using Maximum Likelihood, with 100 bootstrap replicates as described in (Curaba et al., 2019). The list of genes annotated as anthocyanin regulation, biosynthesis and decoration are listed in Supplementary Tables S1–S3, and those were used to search for genes that were differentially expressed and located within the major effect QTLs.

### Real-time quantitative reverse transcription PCR

In order to validate the results of RNA-seq, two candidate genes related to glycosylation and acylation were selected to perform qRT-PCR. First-strand cDNA synthesis was performed on 1 μg of total RNA using the SuperScript™ III First-Strand Synthesis System (Invitrogen, Carlsbad, CA, United States). The reactions were carried out in 10 μl final volume containing 10 ng of cDNA, 5 pmol of each primers, and 5 μl of PowerUp™ SYBR™ Green Master Mix (Applied Biosystems, Foster City, CA, United States). The reactions were run using Roche LightCycler480 Real-time detection system (Roche Diagnostics, Indianapolis, IN) using the following program: 95°C for 2 min, followed by 40 cycles at 95°C for 15 s, 55°C for 15 s, and 72°C for 1 min. Melting curves were analyzed for each primer set. Primers information are provided in Supplementary Table S4. The *UBIQUITIN-CONJUGATING ENZYME* (*UBC28*) was used as the reference gene to calculate the relative expression of the candidate gene by the 2^-ΔCT^ method (Vashisth et al., 2011). The expression ratio was calculated using the ΔΔ-Cycle threshold (CT) method, geometric means (Pfaffl, 2001). Correlation analysis was performed to verify the agreement between qRT-PCT and RNA-seq. Statistical analysis was also performed between the low vs. high glycosylation and acylation groups.

## Results

### Phenotypic variability of anthocyanins, CHA and FQ traits

Extensive phenotypic variation was observed for anthocyanins, CHA and FQ traits for three consecutive years (2017–2019). For FQ traits, 1.5, 2 and 10-fold variation was observed for pH, TSS and TA, respectively (Supplementary Table S5). The variation for individual anthocyanins, CHA and totalANC content (sum of 13 non-acylated and 6 acylated anthocyanins) was also substantial, with more than 14, 10 and 5-fold variation for CHA, most of individual anthocyanins and totalANC, respectively (Supplementary Table S5).

**Figure 1:**
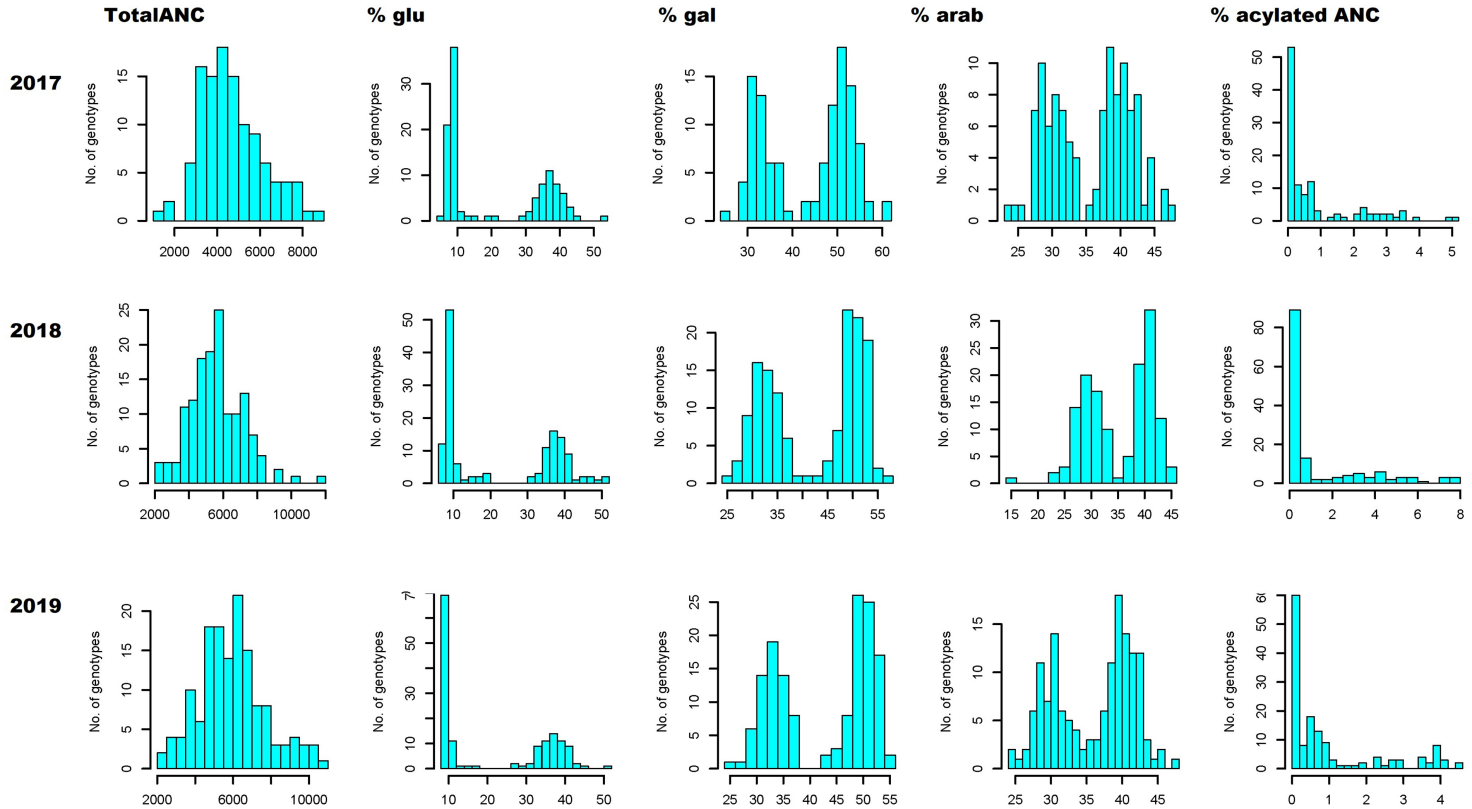
Anthocyanin phenotypic variation. Phenotypic distribution of totalANC (ug/g, fresh weight), contribution (%) of anthocyanins conjugated with different sugar moieties (glucoside, galactoside and arabinoside) and acylated ANC relative to the totalANC, over 3 years (2017–2019). glu, -3-glucoside; gal, -3-galactoside; arab, -3-arabinoside; TotalANC, total anthocyanin.

TotalANC and FQ traits such as pH and TSS followed near-normal distributions, exhibiting a quantitative nature (Figure 1; Supplementary Figure S2), whereas trait distribution of CHA and TA displayed a tendency of skewness toward the lower values (Supplementary Figure S2). Unlike the totalANC, the distribution of acylated and non-acylated anthocyanin concentrations exhibited skewed or bimodal distributions, suggesting oligogenic inheritance of these traits (Figure 1; Supplementary Figure S3).

In total, 19 anthocyanins were identified, which is consistent with previous work (Mengist et al., 2020a,b). The 19 anthocyanins were grouped in different combinations to produce broader summation groups to aid in investigating genotypic differences in anthocyanin concentration and relative composition. The first grouping was based on the aglycone group (e.g., Cyn_glu + Cyn_gal + Cyn_arab) to investigate genotypic differences in aglycone concentration and % contribution of each aglycone group to the totalANC. The results demonstrated that all aglycones exhibited near-normal distribution for both concentration and % contribution data except for Pet. The results from Pet showed a skewed and bimodal distribution for concentration and % contribution, respectively (Supplementary Figures S4a,b). The second grouping was based on sugar moiety to investigate genotypic differences for sugar moiety concentrations and % contribution of each sugar moiety to the totalANC. Both concentrations and % contribution of each sugar moiety exhibited bimodal or skewed distribution (Figure 1; Supplementary Figure S5). Finally, % of acylated anthocyanins (summed up all acylated anthocyanins) to the totalANC was also estimated and the results indicated that acylation data exhibited skewed distribution toward the lower acylation values (Figure 1). Overall, the distribution of the anthocyanin composition based on sugar moiety and acylation suggested that these traits are qualitative and likely under oligogenic inheritance.

**Figure 2:**
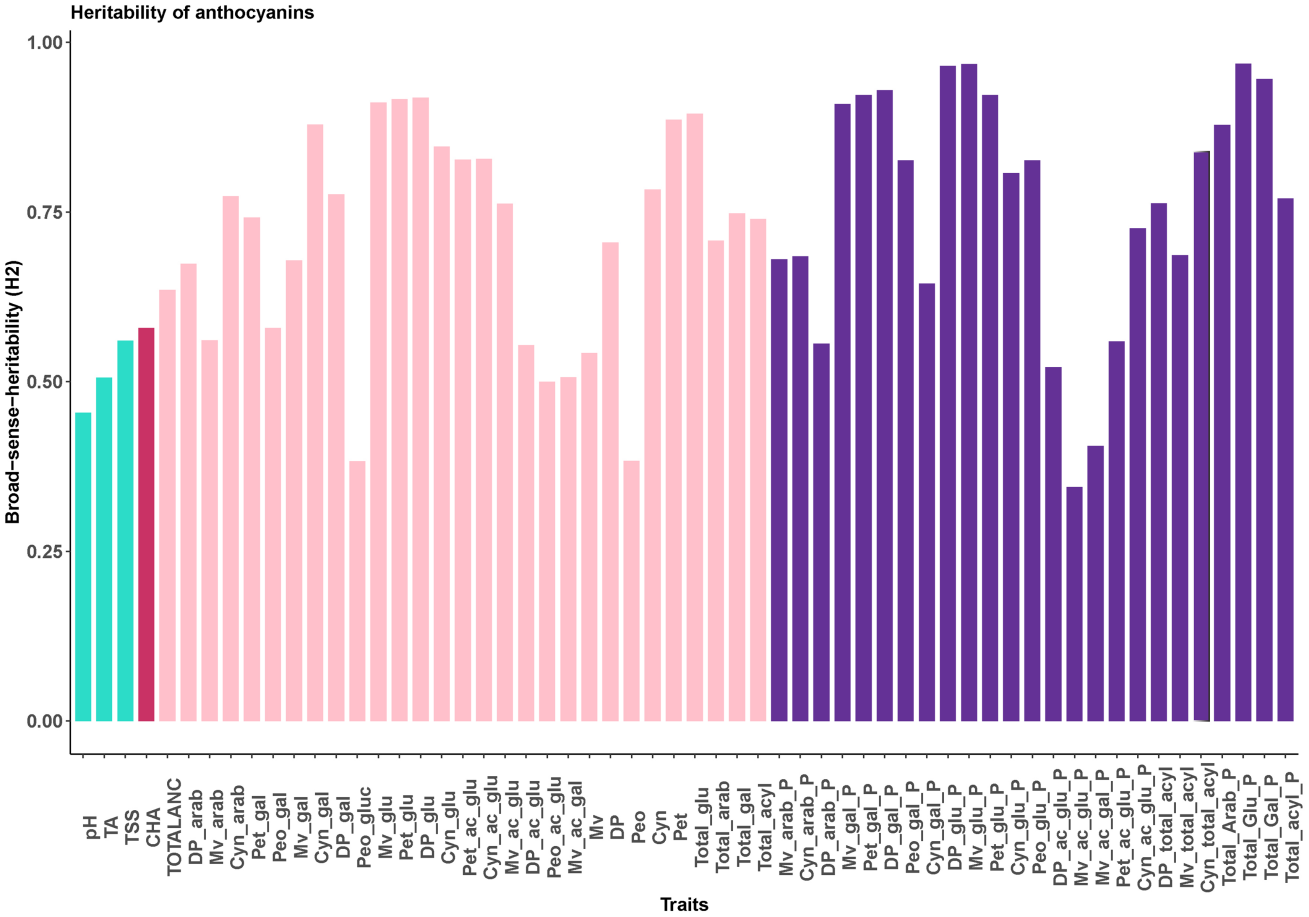
Broad sense-heritability for FQ, CHA and anthocyanin related traits. Dp, delphinidin; Cyn, cyanidin; Peo, peonidin; Mv, malvidin; Pet, petunidin; glu, -3-glucoside; gal, -3-galactoside; arab, -3-arabinoside; ac_glu, -3-(6-acetyl glucoside); ac_gal, -3-(6-acetyl galactoside); _P, relative contribution (%). The bar colors represent fruit quality traits (green), chlorogenic acid concentration (red), anthocyanins concentration (pink), anthocyanins composition (purple).

Broad sense heritability (*H^2^*) estimates revealed that FQ traits, totalANC and CHA are moderately heritable traits, with H^2^ ranging from 45% for pH to 64% for TotalANC (Figure 2). Most of the traits representing individual anthocyanin concentration and composition (e.g., glycosylated, acylated anthocyanin) had high heritability (≥ 70%), indicating these traits are highly heritable traits (Figures 1, 2) and under strong genetic control.

**Figure 3:**
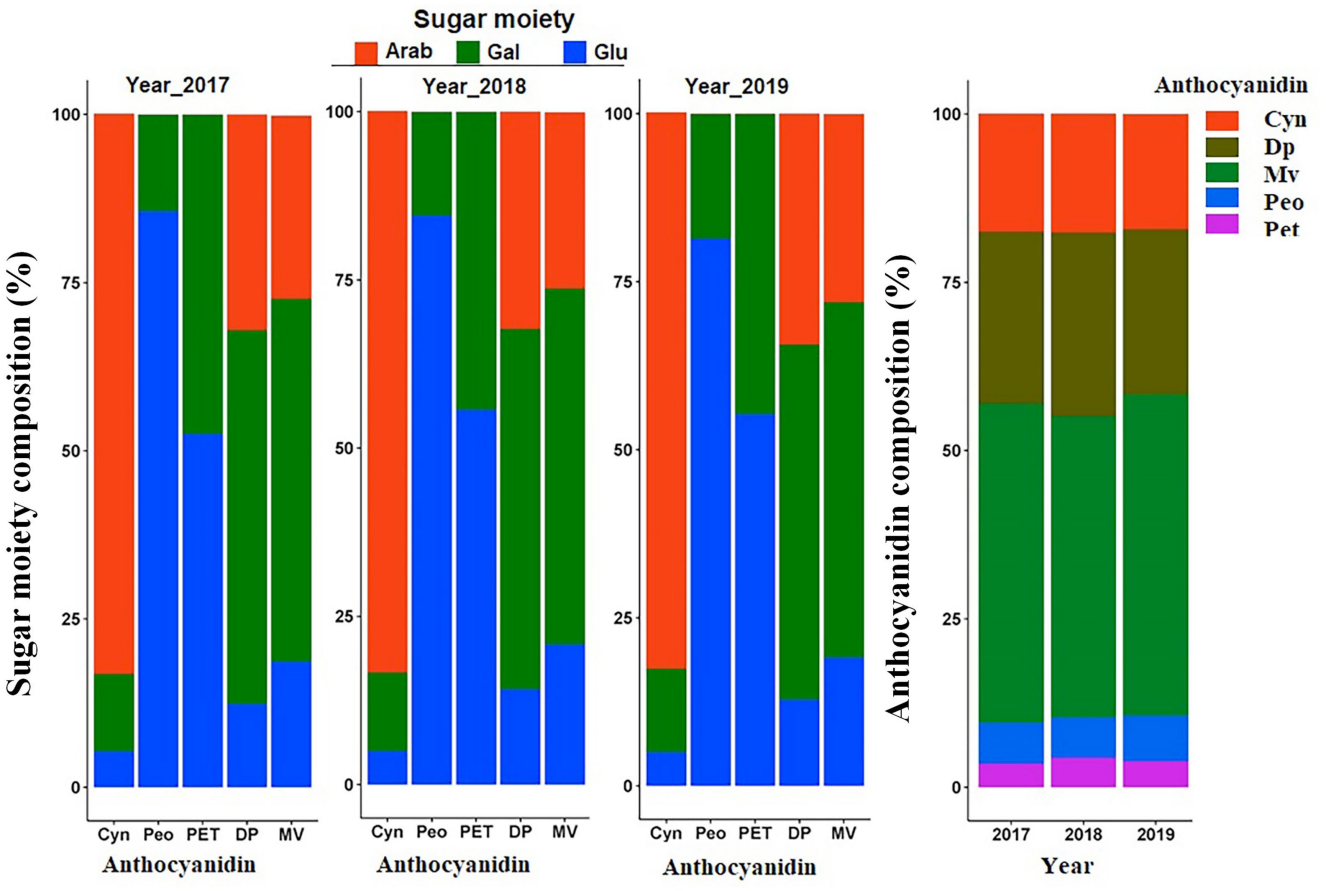
Anthocyanin composition variation. Average composition of each sugar moiety to the respective anthocyanidin group, and average anthocyanidin composition relative to the totalANC in the mapping population over 3 years.

To compare the average composition of anthocyanidins relative to the totalANC, means of the aglycones were computed at mapping population level. Results indicated that Mv followed by Dp were the most abundant anthocyanins. Pet and Peo were the least abundant anthocyanins across 3 years (Figure 3). Similarly, the average composition of sugar moieties indicated that Cyn was a predominantly arab-containing anthocyanin. Peo and Pet core structures conjugated with arab were not detected at all. Instead, a higher portion of the Peo and Pet were conjugated with glu followed by gal sugar moieties. Mv and Dp containing anthocyanins were mainly conjugated with gal followed by arab sugar moieties (Figure 3).

### Association between anthocyanins, CHA and FQ traits

**Figure 4:**
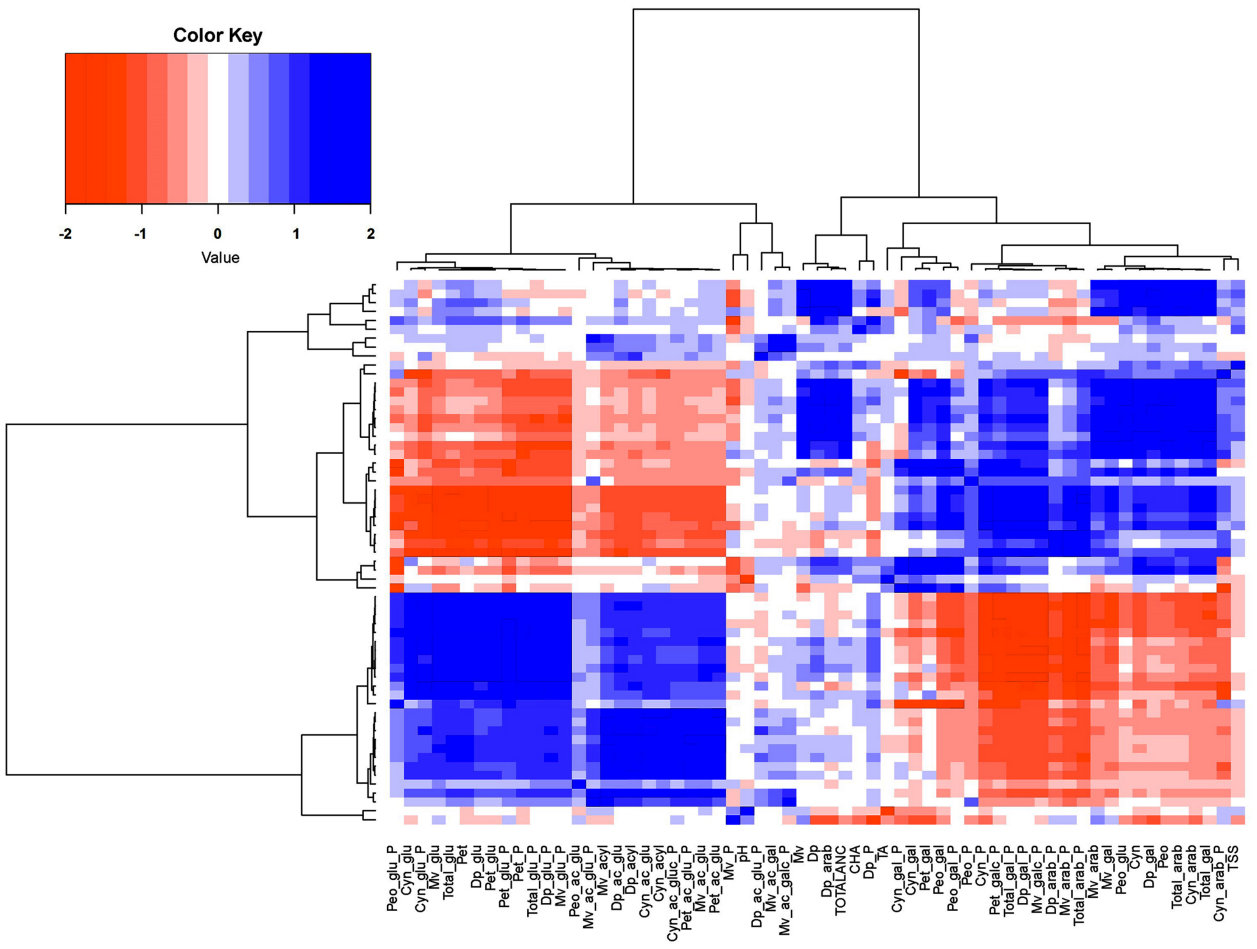
Clustering analysis. Hierarchical clustering analysis of chlorogenic acid concentration, anthocyanins concentration and relative composition (%), and fruit quality traits.

Pearson correlation analysis between anthocyanins, CHA and FQ traits was performed across 3 years (Supplementary Figure S6). As expected, across the 3 years TA was negatively (*p* < 0.05) correlated with pH. TA was positively and significantly (*p < 0.05*) correlated with Cyn-gal, Pet-gal and Dp (Supplementary Figure S6). Another important FQ trait, TSS, exhibited a significant (*p < 0.05*) and positive correlation with arab/gal containing anthocyanins including Total-gal, Total-arab, Cyn-arab, Cyn-gal, Dp-arab, Dp-gal and CHA (Supplementary Figure S6). CHA was significantly (*p* < 0.05) and positively correlated with TotalANC. Since the correlation analysis followed similar patterns across years, the 3 years of data were combined and used to perform a hierarchical clustering (HC) analysis. Results demonstrated that individual anthocyanins clustered based on type of sugar moiety and acylation, suggesting that acylation and glycosylation are the main determinants for the structural diversity of anthocyanins. Furthermore, TSS clustered with arab/gal containing anthocyanins and CHA clustered with total anthocyanin (Figure 4).

### QTL identification for anthocyanins, CHA and FQ traits

For this study, QTL mapping was performed using a linkage map developed from 29 K SNP markers that covered all of the 96 haplotypes (Mengist et al., 2022) and 3 years of phenotypic data. In total, 188 individual QTLs were detected for individual anthocyanins and totalANC concentrations, CHA concentration, anthocyanin composition (% contribution as described above) and FQ traits across 3 years (Supplementary Table S6).

Eight QTLs were identified for FQ traits including four QTLs for TA, two QTLs for pH and two QTLs for TSS (Supplementary Table S6). QTLs regulating both TA and pH were identified on chromosome 3, which are stable across years (3 years for pH, and 3 years for TA) and explained approximately 20% of the phenotypic variance for each trait. As expected from the correlation analysis, the QTLs have an opposite effect between the two traits, increasing pH values causes decreasing TA values. Another QTL controlling TA was identified on chromosome 12 for year-2018. This QTL explained 16% of the phenotypic variance and was associated with increasing TA values (Supplementary Table S6). For TSS, two additional QTLs were identified on chromosomes 8 and 10 for year-2019 and year-2017, and explained 28 and 19% of the phenotypic variance, respectively (Supplementary Table S6).

A QTL controlling CHA concentration was identified on chromosome 2 for three consecutive years (2017–2019). This QTL explained 21, 20, and 19% of the phenotypic variance for 2017–2019, respectively and was associated with increasing CHA values. Analysis of the QTL genotype means using the simple model indicated that a double simplex allele on homologs 1 (h1) of Draper-selection-44392 and 6 (h6) from Jewel were the best allelic configuration for this trait (Table 1). The locus is located between 19 to 29 cM, which corresponds to 8-12 Mb of the diploid blueberry genome, W85_v2 genome on chromosome 2 (Table 1).

**Table 1:** Summary of quantitative trait loci for FQ traits (TSS, TA and pH) anthocyanins concentration and anthocyanin composition detected in the DSxJ mapping population.

Traits	Chr	LOD	*R* ^2^	Position (cM)	Best model	Haplotype	Effect
TSS_2017	10	6.50	19.00	81.00	Double simplex	H2H7	Positive
TSS_2019	8	8.40	23.00	78.86	Double simplex	H2H8	Positive
pH_2018	3	6.50	19.00	83.00	Simplex	H8	Positive
pH_2019	3	7.10	21.00	72.00	Simplex	H8	Positive
TA_2017	3	6.97	22.00	77.95	Simplex	H8	Negative
TA_2018	3	7.49	21.00	98.00	Simplex	H8	Negative
TA_2018	12	5.68	16.00	66.00	Double simplex	H2H8	Positive
TA_2019	3	7.62	22.21	75.00	Simplex	H8	Negative
CHA_2017	2	5.90	21.00	29.00	Simplex	H1H6	Positive
CHA_2018	2	6.90	20.00	19.00	Simplex	H1H6	Positive
CHA_2019	2	6.70	19.00	29.60	simplex	H1H6	Positive
TotalANC_2017	1	6.65	24.00	27.00	Simplex × duplex	H457	Negative
TotalANC_2018	1	7.50	22.00	31.00	Simplex × duplex	H457	Negative
TotalANC_2019	1	7.16	20.00	27.00	Simplex × duplex	H457	Negative
TotalANC_2019	11	7.05	20.00	108.00	Simplex	H8	Positive
TotalANC_2017	12	6.62	23.00	62.00	Double simplex	H16	Positive
Total_arab_2017	4	6.48	22.00	116.00	Simplex	H7	Negative
Total_arab_2018	4	9.86	28.00	114.00	Simplex	H7	Negative
Total_arab_2019	4	8.30	22.50	116.00	Simplex	H7	Negative
Total_gal_2017	4	8.34	29.00	116.00	Simplex	H7	Negative
Total_gal_2018	4	13.22	34.00	114.00	Simplex	H7	Negative
Total_gal_2019	4	10.30	26.81	116.00	Simplex	H7	Negative
Total_glu_2017	4	23.50	61.00	115.00	Simplex	H7	Positive
Total_glu_2018	4	28.00	60.00	115.00	Simplex	H7	Positive
Total_glu_2019	4	25.56	52.36	115.00	Simplex	H7	Positive
Total_arab_2019	11	7.20	16.00	98.00	Simplex	H8	Positive
Total_gal_2019	11	6.80	19.00	95.00	Simplex × duplex	H8	Positive
Total_arab_2017	1	5.06	18.00	26.00	Simplex × duplex	H457	Negative
Total_arab_2018	1	5.24	13.00	30.00	Simplex × duplex	H457	Negative
Total_arab_2019	1	5.60	16.00	36.50	Simplex × duplex	H457	Negative
Total_gal_2019	1	5.36	14.36	36.00	Simplex × duplex	H457	Positive
Arab_P_2017	4	23.00	60.00	115.04	Simplex	H7	Negative
Arab_P_2018	4	19.90	63.00	115.14	Simplex	H7	Negative
Arab_P_2019	4	25.26	55.00	115.98	Simplex	H7	Negative
Gal_P_2017	4	34.38	78.00	115.04	Simplex	H7	Negative
Gal_P_2018	4	42.13	76.00	115.04	Simplex	H7	Negative
Gal_P_2019	4	35.00	68.00	115.97	Simplex	H7	Negative
Glu_P_2017	4	37.00	79.00	115.00	Simplex	H7	Positive
Glu_P_2018	4	43.50	77.00	115.00	Simplex	H7	Positive
Glu_P_2019	4	35.00	65.00	115.97	Simplex	H7	Positive
Acyl_P_2017	2	7.05	25.00	27.00	Simplex	H8	Positive
Acyl_P_2018	2	9.20	26.00	28.90	Simplex	H8	Positive
Acyl_P_2019	2	7.33	20.00	27.25	Simplex	H8	Positive
Acyl_P_2017	4	6.99	24.30	122.00	Simplex	H7	Positive
Acyl_P_2019	4	8.58	24.00	115.00	Simplex	H7	Positive

Chr., chromosome number; LOD threshold values of logarithms of odds; *R*^2^ phenotypic variance explained by the QTL; simplex models are indicated as homologs H1–H4 from Draper-selection-44392 and H5–H8 from Jewel; Glu, -3-glucoside; Gal, -3-galactoside; Arab, -3-arabinoside; _P % contribution; Acyl, acylation; CHA, chlorogenic acid; TSS, total soluble solid; TA, titratable acidity.

In total, 67 QTLs were identified for totalANC and individual anthocyanin concentration on chromosomes 1, 2, 4, 8, 9, 10, 11 and 12, which explained 11 to 67% of the phenotypic variances (Supplementary Table S6). Among them, 34 QTLs overlapped around 115 cM of chromosome 4. These QTLs were responsible for increasing glucoside-based anthocyanins and reducing the arab/gal-based anthocyanins (Supplementary Table S6). Analysis of the QTL genotype means using the simple model indicated that a simplex allele on homolog 7 (h7) from Jewel was the best allelic configuration controlling these traits (Supplementary Table S6). A second hotspot QTL region that was associated with 15 QTLs was identified on chromosome 1, in a region spanning from 18 cM to 35 cM and explaining from 13 to 22% of the phenotypic variance. These QTLs were associated with reducing the concentration of arab and gal containing anthocyanins including Cyn-gal, Mv-arab, Mv-gal, Pet-gal and Peo-gal (Supplementary Table S6). A cluster of six QTLs co-localizing at around 27 cM of chromosome 2, controlled acylation of anthocyanin and explained 14 to 21% of the phenotypic variance (Supplementary Table S6). These QTLs were associated with increasing concentration of acylated anthocyanins including Mv-and Pet-acylated anthocyanins. Analysis of the QTL genotype means using the simple model indicated that a simplex allele on homolog 8 (h8) from Jewel was the best allelic configuration for acylated anthocyanins on this region (Supplementary Table S6). Another cluster of QTLs was identified on chromosome 8, which was associated with increasing Cyn-gal and Pet-gal and reducing Peo-gal concentration values. The peak of the QTLs was located between 64 and 70 cM and explained 19–44% of the phenotypic variance (Supplementary Table S6). Finally, other minor-effect QTLs on chromosomes 9, 10, 11 and 12 were identified. The QTLs were responsible for Mv and total anthocyanin concentration (Supplementary Table S6).

**Figure 5:**
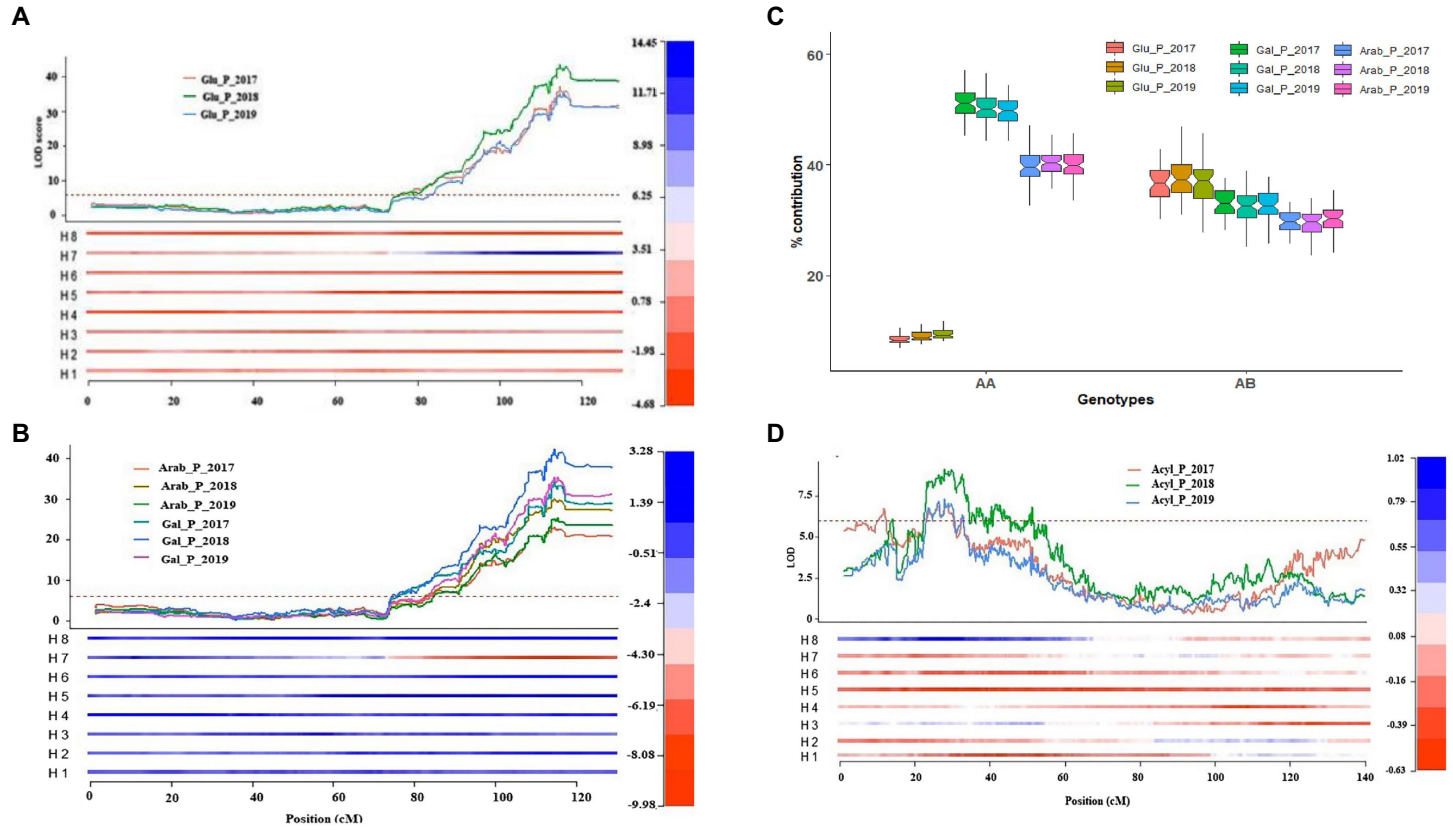
Major-effect QTLs identified for anthocyanin glycosylation and acylation mapped on chromosomes 2 and 4. **(A)** Representation of a major-effect locus mapped on chromosome 4 that increased the amount of glucoside containing anthocyanins; **(B)** Representation of a major-effect locus mapped on chromosome 4 that reduced the amount of galactoside/arabinoside containing anthocyanins; **(C)** Boxplot illustrating the present (AB) and absent (AA) effects of the major – effect locus mapped on chromosome 4 using simplex SNP marker, Chrom04_60364575; **(D)** Representation of a major-effect QTL mapped on chromosome 2 that increased acylation of anthocyanins. The heatmap represents the effect of each homolog relative to the overall phenotypic mean performance. The H1–H8 represents the eight homologs with the first four homologs (H1–H4) inherited from the parent ‘Draper-Selection _44392’ and the other four homologs (H5–H8) inherited from the parent ‘Jewel’.

The % contribution of each anthocyanin to respective anthocyanidin group (e.g., Cyn_glu/Total Cyn) was determined to detect QTLs for the specific anthocyanin composition. In total, 59 QTLs were identified on chromosomes 1, 2, 4, 8, 9 and 11 (Supplementary Table S6). Among them, a cluster of 28 QTLs was identified with a peak at ~115 cM of chromosome 4. These QTLs were regulating the shift between anthocyanin conjugated with glu vs. gal/arab sugar moieties independently from the acylation status (acylated and non-acylated), explained up to 80% of the phenotypic variance and most of the QTLs were stable across years (Supplementary Table S6). As expected, the location and effects of these QTLs coincided with QTLs for individual anthocyanins, which are mainly controlling absolute or relative quantities of glu containing anthocyanins. In fact, the cluster of QTLs on chromosome 4 increase glu-containing anthocyanins thereby increasing the proportion of glu sugar moiety (Supplementary Table S6; Figure 5).

The second largest cluster of QTLs for anthocyanin composition was identified on chromosome 2, accounting for 27% of the phenotypic variance and was associated with increasing acylation of individual anthocyanins (Supplementary Table S6). As expected, these QTLs co-localized with the QTLs for individual acylated anthocyanin concentration (Table 1; Supplementary Table S6). Similar to what was observed for QTLs detected on chromosomes 2 and 4; clusters of QTLs were also identified on chromosomes 1 and 8. The cluster of QTLs on chromosome 1 overlapped with the QTLs identified for anthocyanin concentration, and were responsible for increasing Cyn_arab, and reducing Cyn_gal and Peo_gal. The QTLs explained 18–35% of the phenotypic variance and were stable across 3 years (Supplementary Table S6). The cluster of QTLs on chromosome 8 was responsible for the partitioning between cyanidin gal vs. arab. The QTLs explained 17–37% of the phenotypic variance and were associated with increasing arab and reducing gal (Supplementary Table S6). Interestingly, the cluster of QTLs on chromosome 8 overlapped with the TSS QTL (Supplementary Table S6), suggesting that the QTL for TSS may co-segregate with and/or affect arab sugar metabolism. In addition, two minor-effect QTLs identified on chromosomes 9 and 11 explained 11 and 19% of the phenotypic variance and were associated with increasing the proportion of Cyn_arab (Supplementary Table S6).

QTL analysis was also performed by grouping anthocyanins based on sugar moieties and acylated vs. non-acylated groups and expressed in % relative to the total amount of anthocyanin (e.g., Cyn/_glu + Mv_Glu + Pet_Glu + Peo_Glu + DP_Glu/TotalANC). For % contribution of each sugar moiety, a major-effect QTL was mapped on chromosome 4, which explained up to 80% of the phenotypic variance and was stable across 3 years (Table 1; Supplementary Table S6; Figure 5). The QTL contribute to increasing the proportion of glu and reducing the proportion of arab/gal (Supplementary Table S6). Analysis of the QTL genotype means using the simple model indicated that a simplex allele on homolog 7 (h7) of Jewel was the best allelic configuration (Table 1; Figure 5). Finally, two major QTLs for anthocyanins acylation were mapped on chromosomes 2 and 4. The QTL on chromosome 4 overlapped with the QTL for % contribution of each sugar moiety. The QTL on chromosome 2 peaked ~27 cM. Each QTL explained up to 25% of the phenotypic variance and were associated with increasing anthocyanin acylation in blueberries (Supplementary Table S6). The interval of these QTLs overlap with those of the QTLs detected for acylated anthocyanin concentration.

Overall, the QTL analysis identified overlapping and stable QTLs on chromosome 1, 2, 4 and 8 controlling the conjugations of anthocyanin with the different sugar moieties and acylation grouping, and CHA on chromosome 2 (Supplementary Figure S7). In particular the QTLs on chromosome 4 control the ratio between glucoside vs. galactoside+ arabinoside anthocyanin, the QTL in chr 2 control the ratio between acylated v non-acylated anthocyanin, the QTL on chromosomes 1 and 8 control the ratio between galactoside vs. arabinoside anthocyanin.

### Analysis of candidate genes controlling anthocyanin acylation and glycosylation

In order to identify potential candidate genes involved in regulation of glycosylation and acylation, an RNA-seq experiment was performed using F_1_ genotypes selected from DSxJ mapping population, representing low vs. high glycosylated anthocyanin, and low vs. high acylated anthocyanin (see Materials and methods). A total of 16 strand-specific and pair end cDNA libraries (8 fruit samples) were constructed and sequenced on an Illumina NovaSeq 6000, generating an average 45 million clean reads. Of these, on average 77% of the reads were uniquely mapped to the W85_v2 reference genome sequence, while 16% of the reads were mapped to multiple loci and the remaining reads were excluded due to insufficient read length (Supplementary Table S7). Prior to differential gene expression analysis, data quality was assessed using clustering analysis. The results from clustering analysis indicated that DxJ_232 was an outlier, and it was excluded from the downstream analysis (Supplementary Figure S8).

Gene expression analysis was performed by comparing samples with the following conditions: high-acylation vs. low-acylation; high-glycosylated vs. low-glycosylated. The analysis identified 335 differentially expressed genes (DEGs) in the high vs. low-glycosylated anthocyanin comparison, with 102 and 233 genes being up-and down-regulated, respectively (Supplementary Table S8). Similarly, 460 DEGs were identified between samples representing acylated and non-acylated anthocyanin, with 180 genes up-regulated and the remaining 280 genes down-regulated (Supplementary Table S9). A total of 183 genes were differentially expressed in common in both comparisons/conditions (acylation and glycosylation; Supplementary Figure S9).

**Figure 6:**
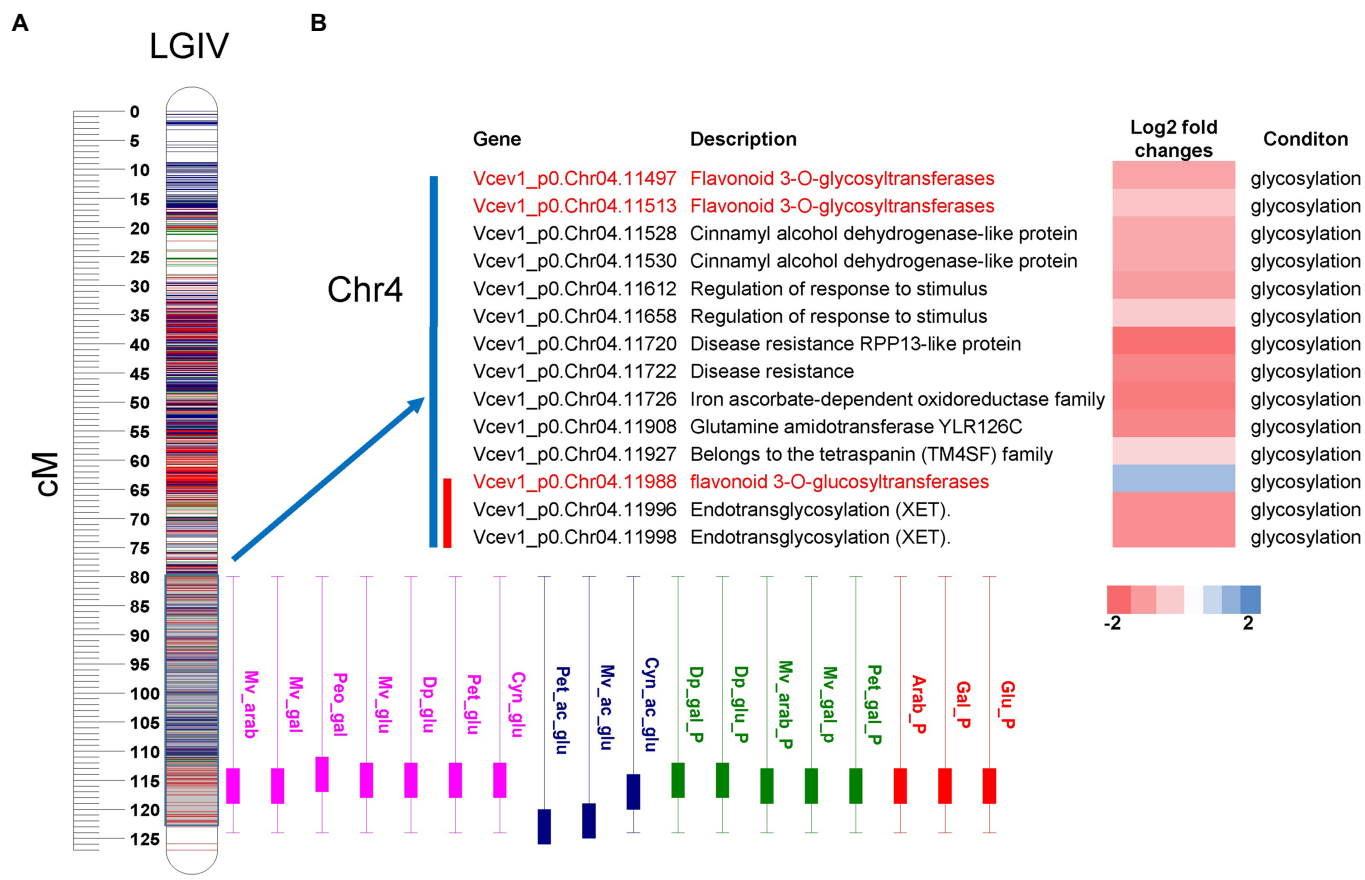
QTL mapping and differentially expressed genes from RNA-seq analysis. **(A)** Representation of the glycosylation related QTLs cluster mapped on chromosome 4. Boxplot represents the QTL position for individual anthocyanin concentrations (rose), acylated anthocyanins (blue), % contribution of individual anthocyanins (green) and % contribution of each sugar moiety to totalANC (red). The boxplot represents the 95% permutation support interval (the interval where the QTL peak exceeds the LOD threshold established using 1,000 permutations and *α* = 0.05). The solid box of the boxplot represents two-LOD support interval. **(B)** List of DEGs located within the genomic region spanning the 95% permutation support interval (blue bar) and two-LOD support interval (red bar). Potential candidate genes are highlighted in red. Log2fold change represents the gene expression levels from down-regulated (red) to up-regulated (blue). Condition represents the following pairwise comparisons performed for gene expression analysis: Glycosylation = high-glycosylated vs. low glycosylated samples. For each trait, the two-LOD and 95% support interval presented here represent the overlap of the QTL regions detected across 3  years.

**Figure 7:**
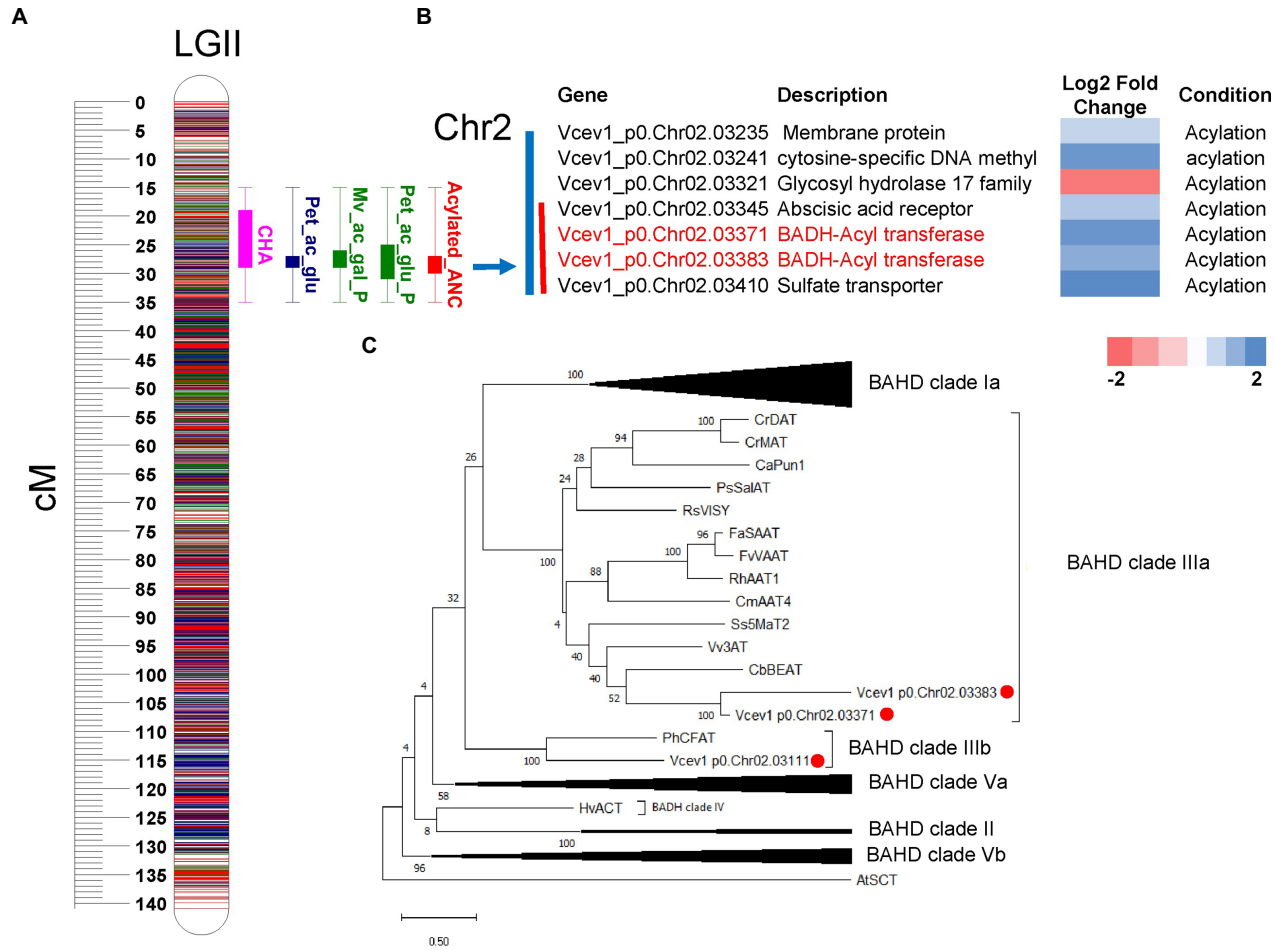
QTL mapping and DEGs from RNA-seq analysis. **(A)** Representation of the acylation related QTLs cluster mapped on chromosome 2. Boxplot represents the QTL position for acylated anthocyanins (blue and green), CHA (rose) and relative contribution (%) of acylated anthocyanin to the totalANC (red). The boxplot represents the 95% permutation support interval (the interval where the QTL peak exceeds the LOD threshold established using 1,000 permutations and *α* = 0.05). The solid box of the boxplot represents two-LOD support intervals. **(B)** List of DEGs located within the genomic region spanning the 95% permutation support interval (blue bar) and two-LOD support intervals (red bar). Genes that are directly related to acylation are highlighted in red. Log2foldchange represents the gene expression levels from down-regulated (red) to up-regulated (blue). Condition represents the following pairwise comparison performed during gene expression analysis: Acylation = high-acylated vs. low-acylated samples. For each trait, the two-LOD and 95% support interval presented here represent the overlap of the QTL regions detected across 3  years. **(C)** Maximum Likelihood phylogenetic tree of selected plant BADH and putative blueberry BADHs identified as DEG within the region spanning the 95% QTL support interval. Bootstrap values are percentage from 100 replicates. The Genbank ID for the sequences are reported in Table S2. Red circles indicate blueberry genes clustered with known BADHs genes.

To search for candidate genes potentially associated with acylation/glycosylation, we performed functional annotation of DEGs and integrated with the QTL regions. To anchor the QTLs to the genome sequence and identify candidate genes, we considered the genomic regions (Mengist et al., 2022) covering the 95% permutation support interval (the interval where the QTL peak exceeds the LOD threshold established using 1,000 permutations and α = 0.05) and two-LOD support interval (narrow-region). For the major-effect locus associated with glycosylation on chromosome 4, 11 DEGs were identified in the 95% permutation support interval, and two of them *Vcev1_p0.Chr4.11497* and *Vcev1_p0.Chr4.11513*, were annotated as UFGT flavonoid 3-O-glycosyltransferases and one *Vcev1_p0.Chr4.11988,* was annotated as UFGT flavonoid 3-O-glucosyltransferase (Figure 6; Supplementary Figure S10). Out of these genes, only three DEGs were identified within the region spanning the two-LOD support interval, including the UDP-glucosyl transferase (*Vcev1_p0.Chr04.11988*) and two endotransglycosylation (*Vcev1_p0.Chr04.11996* and *Vcev1_p0.Chr04.11998*) (Supplementary Tables S1, S8; Figure 6; Supplementary Figure S10). *Vcev1_p0.Chr04.11988* was up-regulated in the samples associated with the dominant effect haplotype controlling high glycosylated anthocyanin and was considered as the best candidate gene controlling this major anthocyanin glycosylation QTL. For the major-effect locus associated with acylation mapped on chromosome 2, seven DEGs were identified in the region spanning the 95% permutation support interval, and four of them were located in the region spanning the two-LOD support interval (Supplementary Table S9; Figure 7). Out of these four genes, two were associated with abscisic acid receptor and the glycosyl hydrolase 17 family and the other two, *Vcev1_p0.Chr02.03371 and Vcev1_p0.Chr02.*03383, were annotated as BAHD-acyltransferases, clade IIIa (Supplementary Table S2; Figure 7) that are known to acylate anthocyanin using p-coumaroyl, feruloyl and caffeoyl-CoA as donor (D’Auria, 2006). The two BADH acyltransferases were both up-regulated, which is consistent with the dominant effect of the haplotype controlling this locus, and these two genes were considered the best candidate genes controlling this locus.

For the QTLs associated with partitioning of low vs. high arab-gal based anthocyanin mapped on chromosome 8 (Cyn-Peo on chr 8; Supplementary Figure S11), 26 and 11 DEGs were identified in the regions spanning the 95% permutation support interval and two-LOD support interval, respectively (Supplementary Table S8; Supplementary Figure S11). Among them, two were annotated as UFGT flavonoid 7-O-glycosyltransferases (*Vcev1_p0.Chr08.20633, and Vcev1_p0.Chr08.20634*) (Supplementary Table S1; Supplementary Figures S10, S11), two as beta-galactosidase (*Vcev1_p0.Chr08.22305, Vcev1_p0.Chr08.22325*) and three as sugar transporter genes (*Vcev1_p0.Chr08.21716*, *Vcev1_p0.Chr08.21718*, and *Vcev1_p0.Chr08.21721*). When examining the candidate genes located in the region spanning the two-LOD support intervals, only the three sugar transporter genes were located within this narrow region (Supplementary Figure S11). Interestingly, this region also overlapped with the TSS QTL. These results suggest that genes involved in the flux of sugars could play a role in the partitioning of arab-gal anthocyanin. However, other genes, like the UFGT located within this region could be considered as candidate genes for future functional characterization.

Finally, for the cluster of QTLs mapped on chromosome 1 associated with low vs. high arab-gal based anthocyanin (Cyn-Peo-Mv, Supplementary Figure S12), 17 and 5 DEGs were identified in the 95% permutation support intervals and two-LOD support interval, respectively (Supplementary Figure S12). Among them, two sugar transporter genes (*Vcev1_p0.Chr01.00504, Vcev1_p0.Chr01.00513*) were located in the 95% permutation support interval and two-LOD support interval, respectively (Supplementary Figure S12). Overall, transcriptome analysis highlighted key genes involved in the glycosylation and acylation of anthocyanins in blueberries.

The expression levels of 2 candidate genes (UFGT-*Vcev1_p0.Chr04.11988* and BADH-*Vcev1_p0.Chr02.03371*) were verified by qRT-PCR. The results indicated that the gene expression level detected by qRT-PCR and RNA-seq analysis were highly correlated (*R*^2^ ≥ 81%). As expected, the expression level of the candidate UFGT gene (*Vcev1_p0.Chr04.11988*) was significantly (*p* < 0.05) higher in the high glycosylated samples, and the expression level of the candidate BADH gene was significantly (*p* < 0.05) higher in the high acylated samples (Supplementary Figure S13).

## Discussion

Multiple human health benefits associated with blueberry consumption drive interest from consumers, food industries and scientific community. In this study, we combined QTL mapping with RNA-seq analysis to dissect the genetic basis of anthocyanins, CHA and FQ traits. A wide phenotypic variation and moderate to high heritability were observed for anthocyanins, CHA and FQ traits in this mapping population. The degree of phenotypic variation and heritability reported in this study for these traits were comparable to that reported in a diverse set of blueberry accessions (Mengist et al., 2020b, 2021) indicating the suitability of this mapping population to explore the genetic basis of these traits. In total, 188 QTLs were identified controlling multiple anthocyanin concentrations and composition, CHA concentration and FQ traits. To our best knowledge, this is the first study to unravel the genetic basis of anthocyanin concentrations, anthocyanin decorations, and CHA concentration in blueberries. Most of these QTLs are concentrated in few genomic regions on chromosomes 1, 2, 4 and 8, which explained phenotypic variation ranging from 13 to 80%. It is important to note that some of the QTLs in this study were not stable across years. The stability of QTLs depends on different factors including sample size of the mapping population and environmental factors that can affect expression of certain genes (Collard et al., 2005). The relatively small sample size (*N* = 196) used in this study may contribute to the instability of these QTLs in addition to the environmental factors (year to year environmental variation).

### Advanced FQ traits genetics and their interaction with anthocyanin genetics

Fruit quality traits affect overall consumer preferences in blueberries (Gilbert et al., 2015). In this study, we identified eight FQ QTLs that explained from 16 to 23% of the phenotypic variance. A TA/pH QTL on chromosome 3, coincides with a previously identified QTL for pH identified at position ~ 38 Mb of the Draper genome of scaffold VaccDscaff9 (Benevenuto et al., 2019; Colle et al., 2019; Mengist et al., 2021). This QTL was stable across 3 years and explained up to 23% of the phenotypic variance and associated with reducing titratable acidity in blueberries (Mengist et al., 2021; Supplementary Table S6). Similarly, the QTL for TSS detected on chromosome 10, coincide with a previously reported TSS QTL, identified at around ~9.4 Mb of the Draper genome of VaccDscaff44 (Colle et al., 2019; Mengist et al., 2021). This QTL was associated with increasing TSS in blueberries (Supplementary Table S6). Overall, these two FQ QTLs, were detected in different genetic backgrounds and could be targets for candidate gene analysis and marker-assisted selection in blueberries. Interestingly, TSS was positively correlated with galactoside/arabinoside containing anthocyanin and the TSS QTL mapped on chromosome 8 overlapped with a glycosylation QTL, suggesting that these traits may be have a shared genetic control (see below for more discussion).

### First insight into CHA genetics and its relation with anthocyanin

This is the first study to evaluate the genetic basis of genotypic difference for CHA concentration in blueberries. This study identified a stable QTL on chromosome 2, associated with increasing CHA concentration and located similar genomic region of acylated anthocyanins QTLs (Supplementary Table S6). At phenotypic level, we did not observe statistically significant correlation between acylated anthocyanins and CHA (Supplementary Figure S6).Similar observations were reported in previous studies (Yousef et al., 2016; Mengist et al., 2020b). Analysis of the QTL genotype means using simple model indicated that a double simplex marker (H1H6) on homolog 1 (H1) of DS-44392 and homolog 6 (H6) of Jewel, and a simplex marker (H8) on homolog 8 (H8) of Jewel were the best model for CHA QTL and acylated anthocyanin QTLs, respectively (Supplementary Table S6). These results indicated that the QTLs for CHA and acylated anthocyanin were located in different haplotypes (Supplementary Table S6). In this condition, the probability of an F_1_ genotype carrying all three haplotypes (H1H6H8) are relatively low. Assuming a bivalent pairing and a tetraploid individual, we expect a total of 36 genotypes and 3/36 of the genotypes carrying all three haplotypes (Hackett et al., 2013; Mengist et al., 2018). Therefore, this observation could explain why, besides been controlled by a QTL spanning the same regions, the two traits are not correlated. Our results also highlighted that CHA was significantly and positively correlated with total anthocyanin, which is consistent with previous studies in blueberry (Yousef et al., 2016; Mengist et al., 2020b). This observation imply that in blueberry it will be possible to simultaneously select for both high CHA and anthocyanin concentration.

### The genetic basis of total anthocyanin is quantitative in nature

Understanding the genetic determinants of anthocyanin concentrations will facilitate development of new cultivars with higher totalANC. A near-normal distribution of totalANC observed in this mapping population suggests that totalANC may be controlled by multiple and minor-effect genes. Consistent with the phenotypic data distribution, we did not identify stable and major-effect QTLs for totalANC, indicating that totalANC in this mapping population is polygenic and affected by environmental conditions (Jaakola, 2002, 2013; Stevenson and Scalzo, 2012; Scalzo et al., 2015; Mengist et al., 2020a,b). Consistent with this result, previous studies reported that totalANC in blueberry is normally distributed and under environmental control (Stevenson and Scalzo, 2012; Scalzo et al., 2015; Mengist et al., 2020a,b). Anthocyanin accumulation is a result of successive enzymatic reactions, which are controlled by structural and regulatory genes. The structural genes encode enzymes including chalcone isomerase (CHI), flavonoid 3-hydroxylase (F3H/FHT), flavonoid 3′-hydroxylase (F3′H), dihydroflavonol-4-reductase (DFR), anthocyanidin synthase (ANS), and 3-glycosyltransferase (3-GT) and O-methyltransferase (OMT) enzymes, which are responsible for the sequential enzymatic reactions. Multiple regulatory genes such as MYB, basic Helix Loop Helix (bHLH) and WD40-repeat proteins, regulate the expression of the structural genes, and the expression of these transcription factors is under environmental control (Jaakola, 2002, 2013; Matsuda et al., 2015). These interconnected enzymes and regulatory mechanism along with environmental factor likely contribute to the polygenic nature of this trait, and its lower hereditability, which limit potential for selection (Jaakola, 2002, 2013; Matsuda et al., 2015; Colle et al., 2019; Mengist et al., 2020b).

### A few genes govern glycosylation and acylation of anthocyanins in blueberry

Glycosylation is a prominent modification reaction and often the final step in the flavonoid biosynthetic pathway. Anthocyanidin glycosylation is essential for the conversion into anthocyanins and contributes to stability and transport of anthocyanins to the vacuole (Vogt and Jones, 2000; Jaakola, 2002, 2013; Zifkin et al., 2012). The glycosylation of anthocyanins involves the transfer of nucleotide–diphosphate-activated sugar to low molecular weight substrate *via* the glycosyltransferases. The activated sugar form includes UDP-glucose, UDP-galactose, UDP-arabinose and UDP-rhamnose (Ford et al., 1998; Vogt and Jones, 2000; Vorsa and Polashock, 2005). In this study, three major-effect QTLs were identified on chromosomes 1, 4 and 8 controlling anthocyanin glycosylation. The QTL on chromosome 4 was stable across 3 years and explained up to 80% of the phenotypic variance. The QTL genotype mean analysis indicated a simplex SNP marker (H7) from the parent ‘Jewel’ was the best fit for the QTL model. The presence/absence of the QTL at the region associated with this haplotype determines the amount of glucose containing anthocyanins in this mapping population. The presence of a simplex SNP allele causes a significant increment on glucoside containing anthocyanins and only a slight decrease on arabinose/galactose containing anthocyanins (Figure 5C). A candidate gene, a *flavonoid 3-O-glucosyltransferas*e, was identified within this QTL region. This gene was up-regulated in the set of samples with high-glucosylated anthocyanin compared to the set of sample with low-glucosylated anthocyanin, suggesting that the difference in anthocyanin UDP-glucose formation in this mapping population could be dependent on of the anthocyanin 3-O-glucosyltransferase allele. These results also suggest that the gene encodes a glycosyltransferase enzyme that is highly specific for the UDP-glucose. Consistent with this hypothesis, earlier evidences suggested that the UFGTs have broad substrate specificity, but exert regioselectivity and regiospecificity in many cases including sugar donor (Ford et al., 1998; Vorsa et al., 2003; Vorsa and Polashock, 2005). For example, in *V. vinifera*, anthocyanidins can only be O-glycosylated at the C3 position with the addition of glucose by the activity of UFGTs but did not exhibit activity with UDP-galactose (Ford et al., 1998). In cranberry, a close relative of blueberry, the difference in anthocyanin glycosylation detected between two cranberry species was a function of different anthocyanin 3-O-glycosyltransferase alleles (Vorsa et al., 2003; Vorsa and Polashock, 2005). Overall, it is possible to hypothesize that blueberries have more than one glycosyltransferase enzymes that use specific sugar donors.

The other two QTLs on chromosomes 1 and 8 specifically control the galactoside containing anthocyanins. This suggests the presence of a variety of UFGTs specific to sugar donors including UDP-galactose and UDP-arabinose (Ford et al., 1998; Vogt and Jones, 2000; Vorsa et al., 2003; Vorsa and Polashock, 2005). Two 7-O-Glycosyltransferases were differentially expressed in the QTL region mapped on chromosome 8. Within the QTL region, three sugar transporter genes were also differentially expressed between the high-and low–galactoside anthocyanins. Given the positive and significant correlation between the TSS and galactoside/arabinoside and an overlapping stable QTL for TSS mapped on the same region of chromosome 8, it is possible that three-sugar transporter genes may regulate the flux of sugar molecules available for the glycosylation reaction and indirectly control the glycosylation reaction at this locus. Similarly, for the major-effect QTL mapped on chromosome 1, the RNA-seq analysis did not identify any glycosyltransferases genes in this region. Instead, two sugar transport genes were differentially expressed between the high and low-galactoside group in the QTL region. Although we did not detect any QTL for TSS in this region, we did not exclude the sugar transporter family as candidate genes because of the positive association between TSS and arabinoside/galactoside. Similar findings indicating that TSS has a significant and positive correlation with arabinoside/galactoside containing anthocyanins in blueberries were previously reported (Mengist et al., 2020a,b).

Acylation of anthocyanins is the process of adding an acyl group to the anthocyanins, which increases color stability, reduces their sensitivity to pH change, sulfite bleaching, and thermal degradation (Giusti and Wrolstad, 2003) and could be involved in bioaccessibility and bioavailability of anthocyanins (McDougall et al., 2007; Correa-Betanzo et al., 2014; Wiczkowski et al., 2016; Mengist et al., 2020a). In this study, the two major-effect loci identified on chromosomes 2 and 4 control 50% of the phenotypic variation in this mapping population. The acylation QTL on chromosome 4 overlaps with the glucosylation QTL, but the annotation and gene expression analysis did not identify any acyl-transferase enzymes in this QTL region. These results suggest that the glucosylation QTL may affect the acylation of anthocyanin. Consistent with this hypothesis, it is important to note that glycosylation and acylation are successive enzymatic reactions, with glycosylation preceding the acylation reaction. In addition, acylated anthocyanins detected in this mapping population are predominantly UDP-glucose anthocyanins, and the glucosylation QTL on chromosome 4 is specific to UDP-glucose anthocyanins. Therefore, the glucosylation QTL which increases UDP-glucoside anthocyanins could also increase the availability of the anthocyanin substrate used for acylation and thereby affecting anthocyanin acylation (Zifkin et al., 2012; Jaakola, 2013; Mengist et al., 2020b). Thus, the detection of an acylation QTL on chromosome 4 could be an indirect effect of the glucosylation QTL.

The acylation QTL mapped on chromosome 2 is independent of glycosylation. The QTL genotype mean analysis indicated a simplex SNP marker (H8) from the parent ‘Jewel’ was the best fit for the QTL model. The presence of the QTL allele at this haplotype region increases acylation of anthocyanins in this mapping population. Two candidate genes, BADH acyltransferases, were identified in this QTL region. The two genes were up-regulated in samples with high acylated anthocyanin. The BAHD acyltransferase family catalyze the acylation of many plant secondary metabolites (D’Auria, 2006; Rinaldo et al., 2015). BAHD acyltransferases transfer an acyl-activated CoA thioester donor to an acceptor molecule (D’Auria, 2006). The phylogenetic analysis demonstrated that these two BAHD acyltransferases clustered with BAHD clade IIIa (Supplementary Figure S14). This group includes *anthocyanin 3-o-glucoside-6″-o-acyltransferase* (Vv3AT), and *malony-CoA:anthocyanin 5-glucoside 4″’-O-malonyltransferase* (SsMat2), which are responsible for acylation of anthocyanins in *Vitis vinifera* and *Salvia splendens,* respectively (Suzuki et al., 2004; Tuominen et al., 2011; Rinaldo et al., 2015). Thus, the two BADH acyltransferase genes are most likely the putative candidate genes for acylation of anthocyanins in blueberry.

### Implications of acylation and glycosylation QTLs for blueberry breeding

Interest in anthocyanin concentration and diversity has increased over the past decades, due to the health effect associated with this bioactive compound, which drives interest from consumers, the food industry and the scientific community. Development of cultivars with high-health promoting phytochemicals and superior FQ traits is a breeding priority for blueberry (Edger et al., 2022). Traditional blueberry breeding is a time consuming (10–15 years), expensive, and laborious process. In addition, integrating metabolite traits in routine selection has not been feasible for most breeding programs, as phenotyping metabolites demands technical skills and is expensive and tedious. Therefore, the current blueberry-breeding programs largely concentrate their efforts on traditional traits including FQ traits. To fill the current gaps of blueberry breeding and to meet the consumers demand, it is useful to integrate traditional breeding with marker-assisted selection to speed up the cultivar development process and reduce the costs for developing superior cultivars.

To establish marker–assisted selection strategy, the first step is to understand the genetic basis of the trait of interest, and if possible, identification of major and stable QTLs that will be used for marker-assisted selection. In this study, we provide the first insight into the quantitative nature of anthocyanin concentration, which limits the development and use of marker-assisted selection for this trait (Collard et al., 2005; Collard and Mackill, 2008). However, the major and stable QTLs identified for acylation and glycosylation are promising, and represent a foundational work to develop, validate and implement marker assisted selection for these traits (Collard et al., 2005; Collard and Mackill, 2008; Mengist et al., 2018).

For glycosylation of anthocyanin, a few studies highlighted the possible role of glycosylation in bioaccessibility/bioavailability (Hollman et al., 1999; Stintzing et al., 2002; Frank et al., 2003; Khoo et al., 2017; Mengist et al., 2020a). For instance, earlier studies (Hollman et al., 1999; Stintzing et al., 2002; Frank et al., 2003; Khoo et al., 2017) reported that glycosylated anthocyanins are more bioavailable in grapes, wines and natural pigments. However, recently, our study (Mengist et al., 2020a) reported that sugar moiety did not exhibit consistent and significant effect on bioaccessibility of anthocyanins though there was a slight increase in bioaccessibility of glucose containing anthocyanins.

Considering the multiple uses of blueberry fruits ranging from fresh consumption to source of anthocyanin as a natural colorant, anthocyanin acylation is an important trait. Indeed, acylated anthocyanins are more stable when used as natural colorant. In addition, acylated anthocyanins affect their nutrigenomic properties like bioaccessibility and/or bioavailability (Kurilich et al., 2005; Charron et al., 2009; Mengist et al., 2020a). In fact, few *in vitro* bioaccessibility studies observed that acylation increases the bioaccessibility of anthocyanins in blueberries and red cabbage (McDougall et al., 2007; Correa-Betanzo et al., 2014; Mengist et al., 2020a). This effect could be due to higher stability of acylated ANC to gastrointestinal conditions of elevated pH (McDougall et al., 2007; Correa-Betanzo et al., 2014; Mengist et al., 2020a). Although no direct evidence has reported the effect of acylation on anthocyanin bioavailability in blueberries, studies in other crops such as sweet potato and red wine (Oliveira et al., 2019), and red cabbage (Wiczkowski et al., 2016) demonstrated that acylated anthocyanins have higher bioavailability than non-acylated anthocyanins. Overall, this study provides valuable information for future fine mapping, functional gene analysis, and marker-assisted development and selection. Thus, selection for acylation of anthocyanins could lead to increased delivery of these health-related bioactives.

## Data availability statement

RNA-seq datasets are available under NCBI accession: PRJNA842250. https://www.ncbi.nlm.nih.gov/sra/PRJNA842250. The linkage map was made available through the genome database for vaccinium (GDV), https://www.vaccinium.org/bio_data/1659687. Genotypic and phenotypic data are made available in the supplementary data (1-phenotypic data, 2-molecular markers).

## Author contributions

MI, ML, and MF: funding acquisition and project administration. MI and MM: conceptualization, formal analysis, writing—original draft preparation, and visualization. MI, MM, and MG: methodology. MM, MG, and BM: investigation. NB, CL, and TM: resources. MM, MI, and BP: data curation. ML, MF, MG, TM, BM, BP, NB, CL, and MF: writing—review and editing. All authors contributed to the article and approved the submitted version.

## Funding

This research was supported by the Foundation for Food and Agriculture Research (FFAR) under award number 534667. MI, MM, and ML were also supported by the United States Department of Agriculture National Institute of Food and Agriculture, Hatch project 1008691, and the National Institute of Food and Agriculture, United States Department of Agriculture, under award number 2019–51181–30015, project “VacciniumCAP: Leveraging genetic and genomic resources to enable development of blueberry and cranberry cultivars with improved fruit quality attributes.” We dedicate this article in Chad Finn’s memory. Chad Finn served as a world-renowned blueberry breeder and released numerous blueberry cultivars as part of the USDA-ARS and Oregon State University. As a passionate geneticist and resourceful collaborator, Finn made significant contributions to advance genetic discoveries in this crop. The work presented here represent an example of its collaborative contributions to the discipline. Many thanks to the German network for bioinformatics infrastructure (de.NBI, grant 031A533A) and the Bioinformatics Resource Facility (BRF) at the Center for Biotechnology (CeBiTec) at Bielefeld University for providing an environment to perform the computational analyses.

## Conflict of interest

The authors declare that the research was conducted in the absence of any commercial or financial relationships that could be construed as a potential conflict of interest.

## Publisher’s note

All claims expressed in this article are solely those of the authors and do not necessarily represent those of their affiliated organizations, or those of the publisher, the editors and the reviewers. Any product that may be evaluated in this article, or claim that may be made by its manufacturer, is not guaranteed or endorsed by the publisher.

## Supplementary material

The Supplementary Material for this article can be found online at: https://www.frontiersin.org/articles/10.3389/fpls.2022.964656/full#supplementary-material
