# Supplementary material for: Dissecting the genetic basis of bioactive metabolites and fruit quality traits in blueberries (Vaccinium corymbosum L.)
Source: Front Plant Sci. 2022 Sep 2;13:964656. doi: 10.3389/fpls.2022.964656 (PMC9478557; doi:10.3389/fpls.2022.964656)
Supplement: Supplementary file 3 [file Data_Sheet_3.docx]

**Dissecting the genetic basis of bioactive metabolites and fruit quality traits in blueberries (Vaccinium corymbosum L).** Mengist et al.


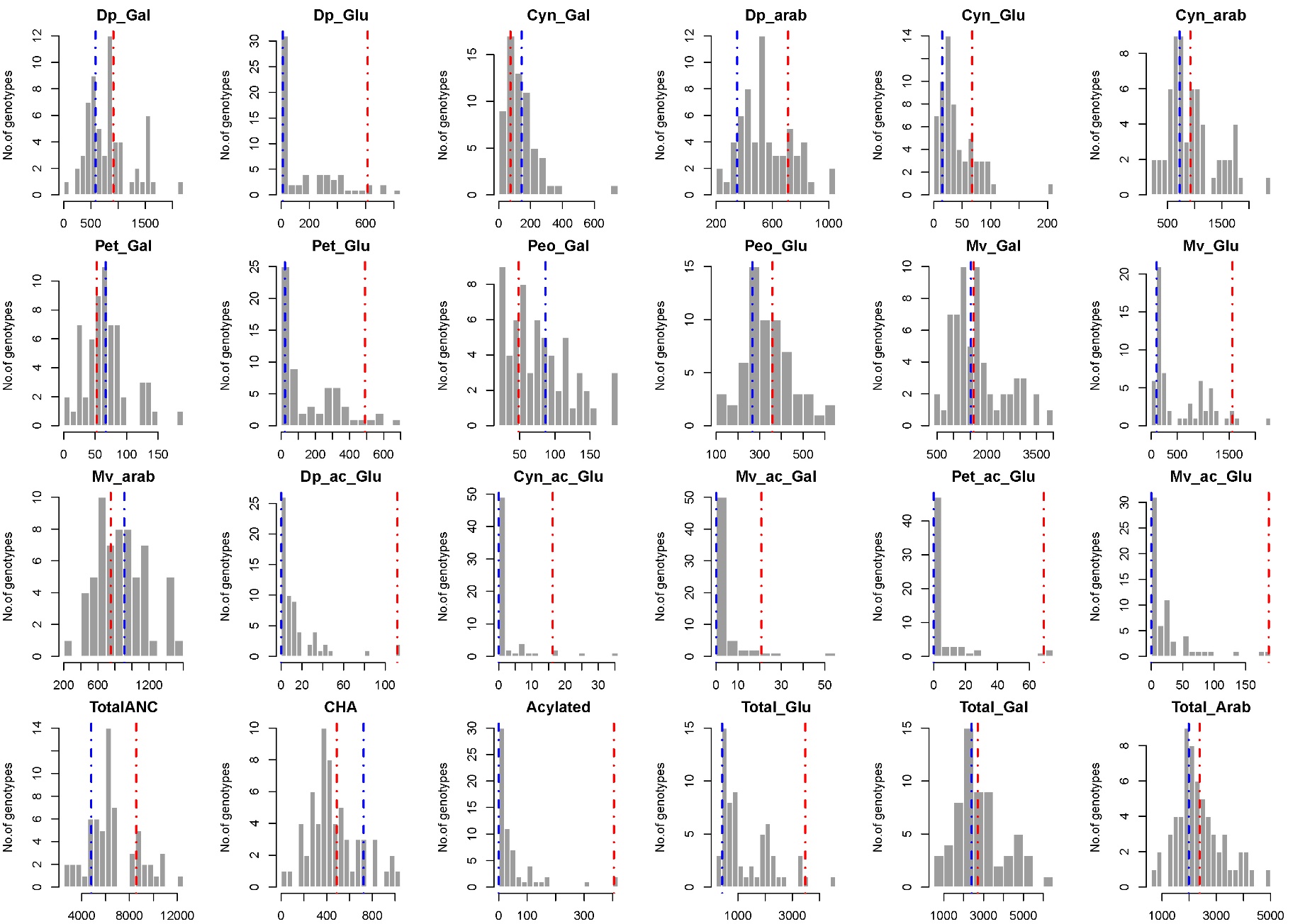


**Figure S1**. **Phenotypic distribution of anthocyanin and CHA concentration** (**ug/g, fresh weight) 60 F_1_ genotypes and the two parents. The red and blue dotted lines refer the parents Draper-44392 and Jewel, respectively.**


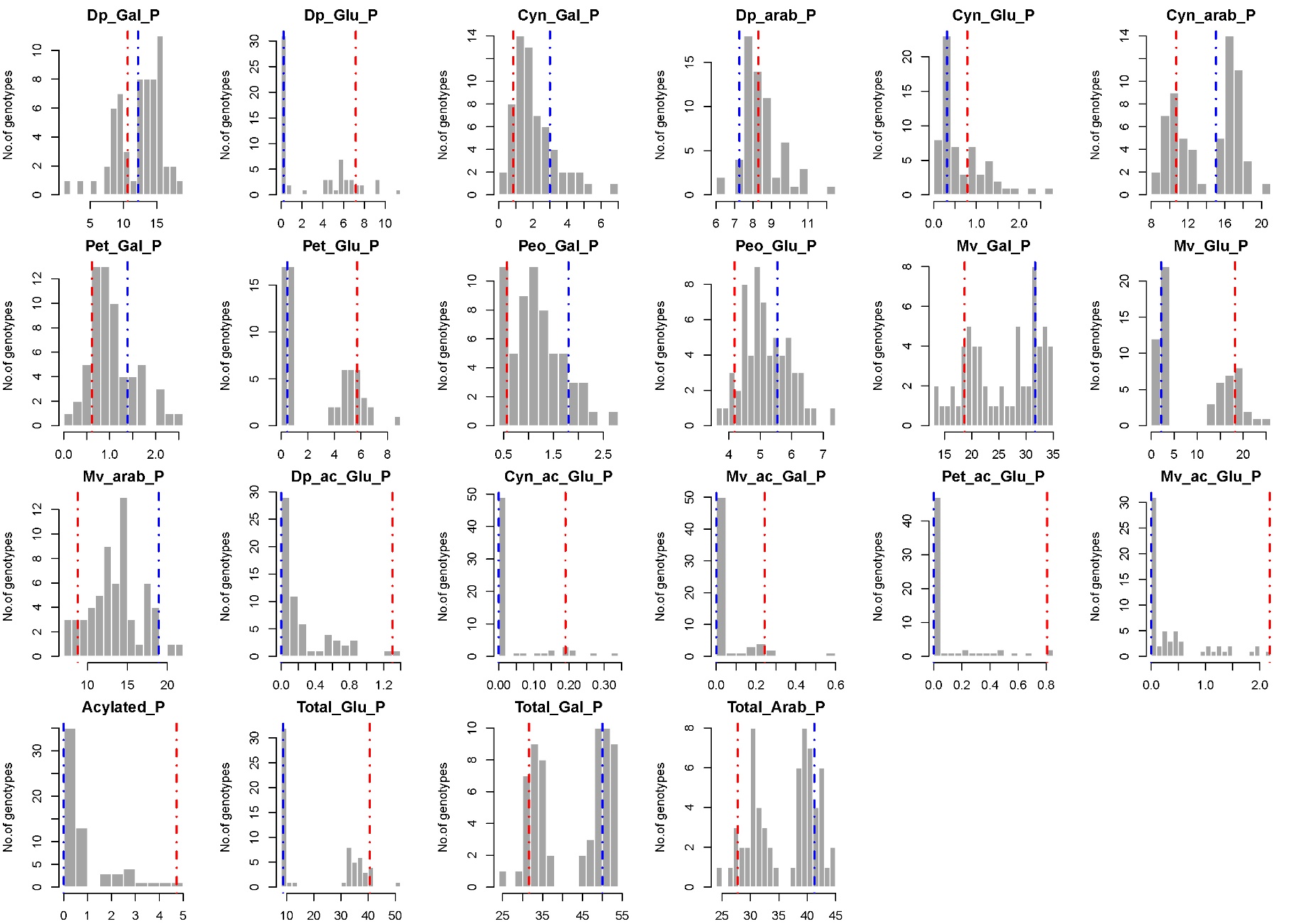


**Figure S1 continue**. **Phenotypic distribution of anthocyanin composition** (**%) of 60 F_1_ genotypes, and the two parents. The red and blue dotted lines refer the parents Draper-44392 and Jewel, respectively.**


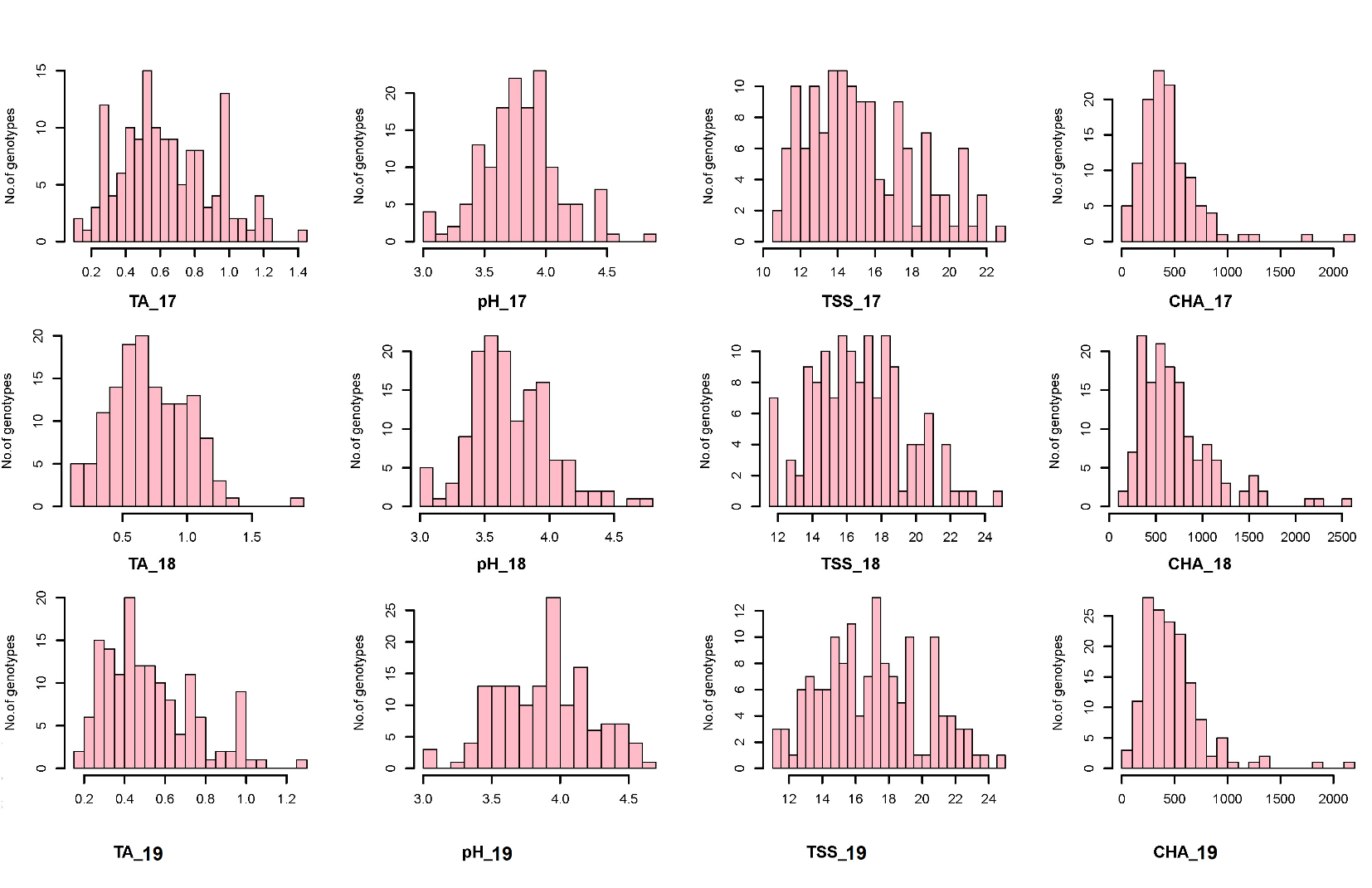


**Figure S2. Phenotypic distribution of chlorogenic acid concentration** (**ug/g, fresh weight) and fruit quality traits over three years.**


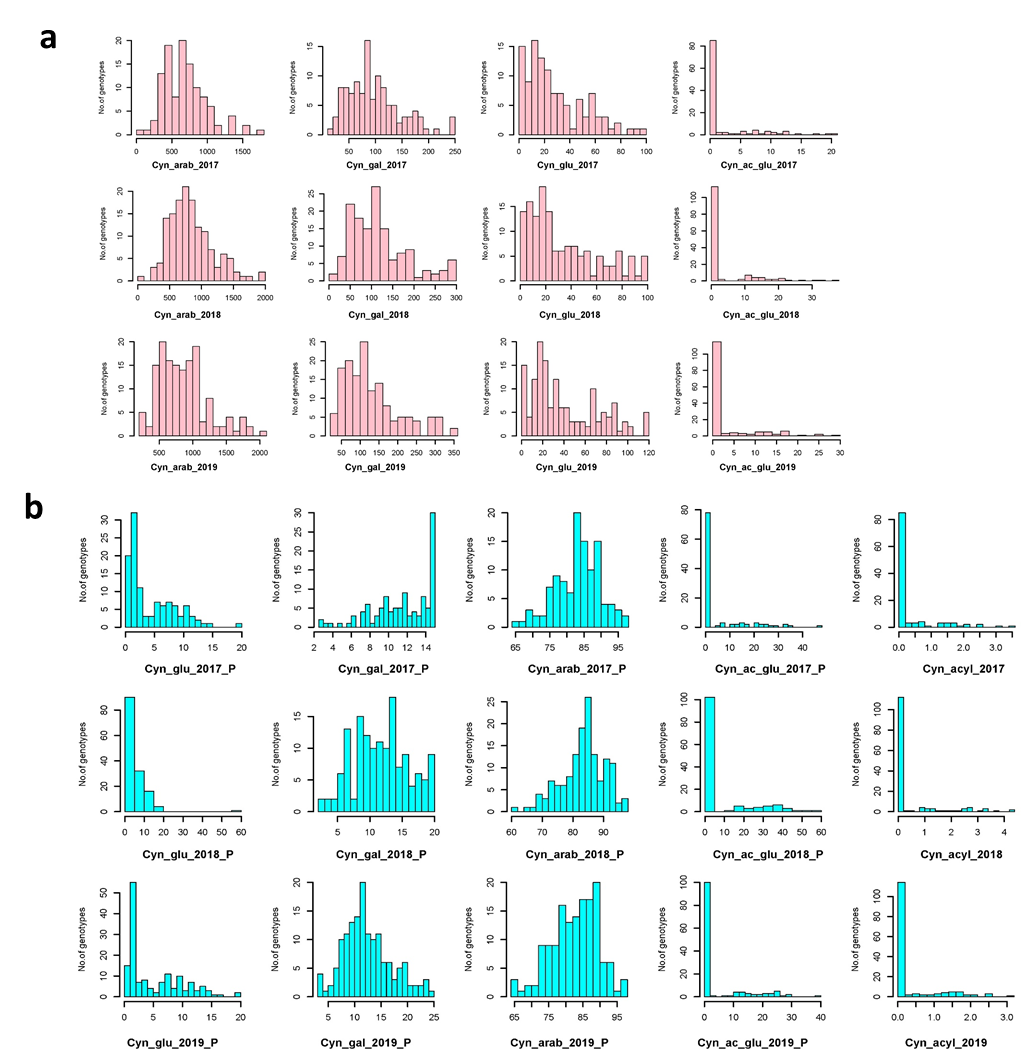


**Figure S3. Phenotypic distribution of concentration** (**ug/g, fresh weight) and relative contribution (%) of anthocyanin over three years. a) Concentration; b) relative contribution (%) to the total cyanidin group.**


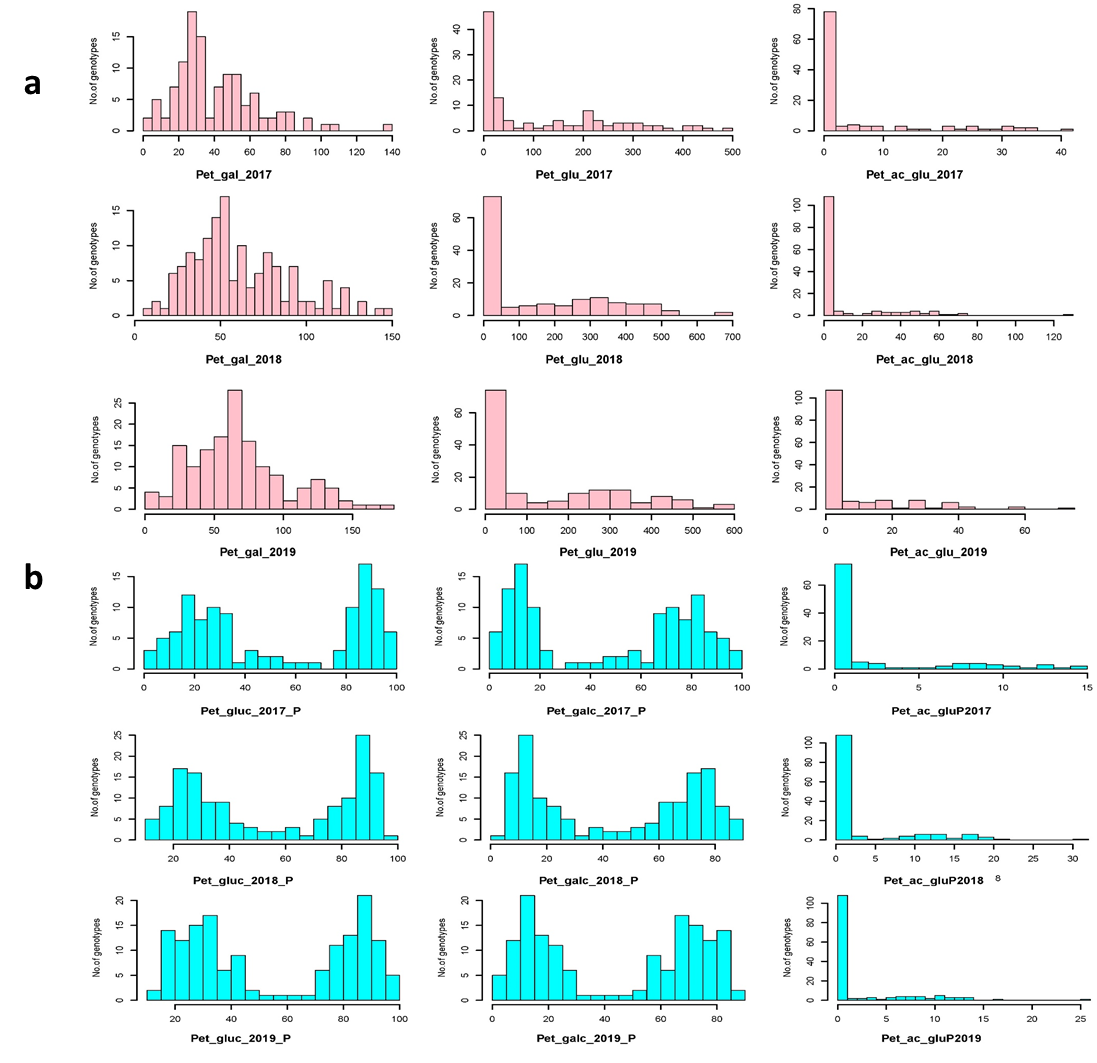


**Figure S3 continue. Phenotypic distribution of concentration** (**ug/g, fresh weight) and relative contribution (%) of petunidin aglycone based anthocyanins traits over three years. a) concentration; b) relative contribution (%) to the petunidin group. FW, fresh weight.**

**
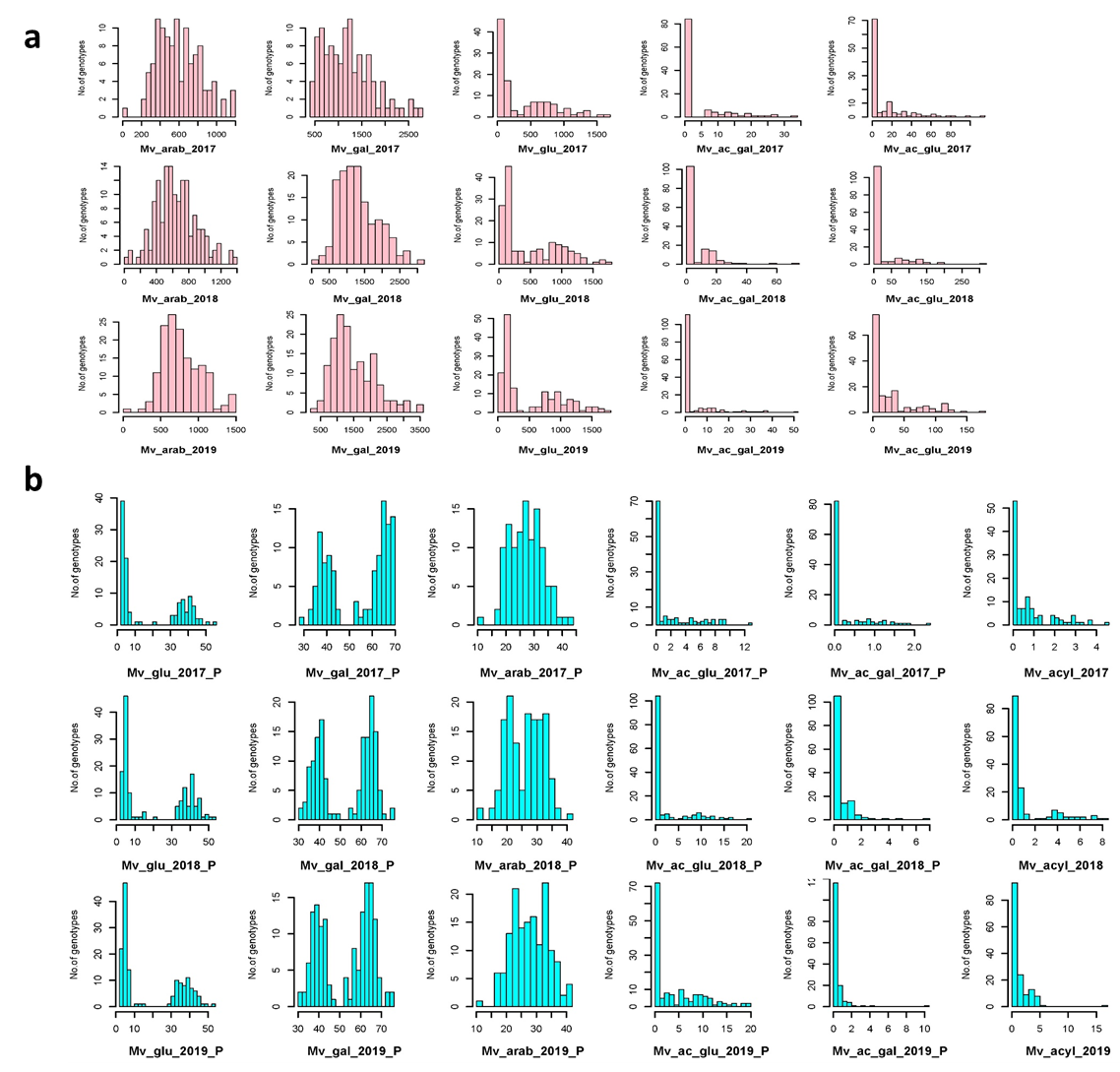
**

**Figure S3 continue. Phenotypic distribution of concentration** (**ug/g, fresh weight) and relative contribution (%) of malvidin aglycone based anthocyanins traits over three years. a) Concentration; b) relative contribution (%) to the malvidin group.**


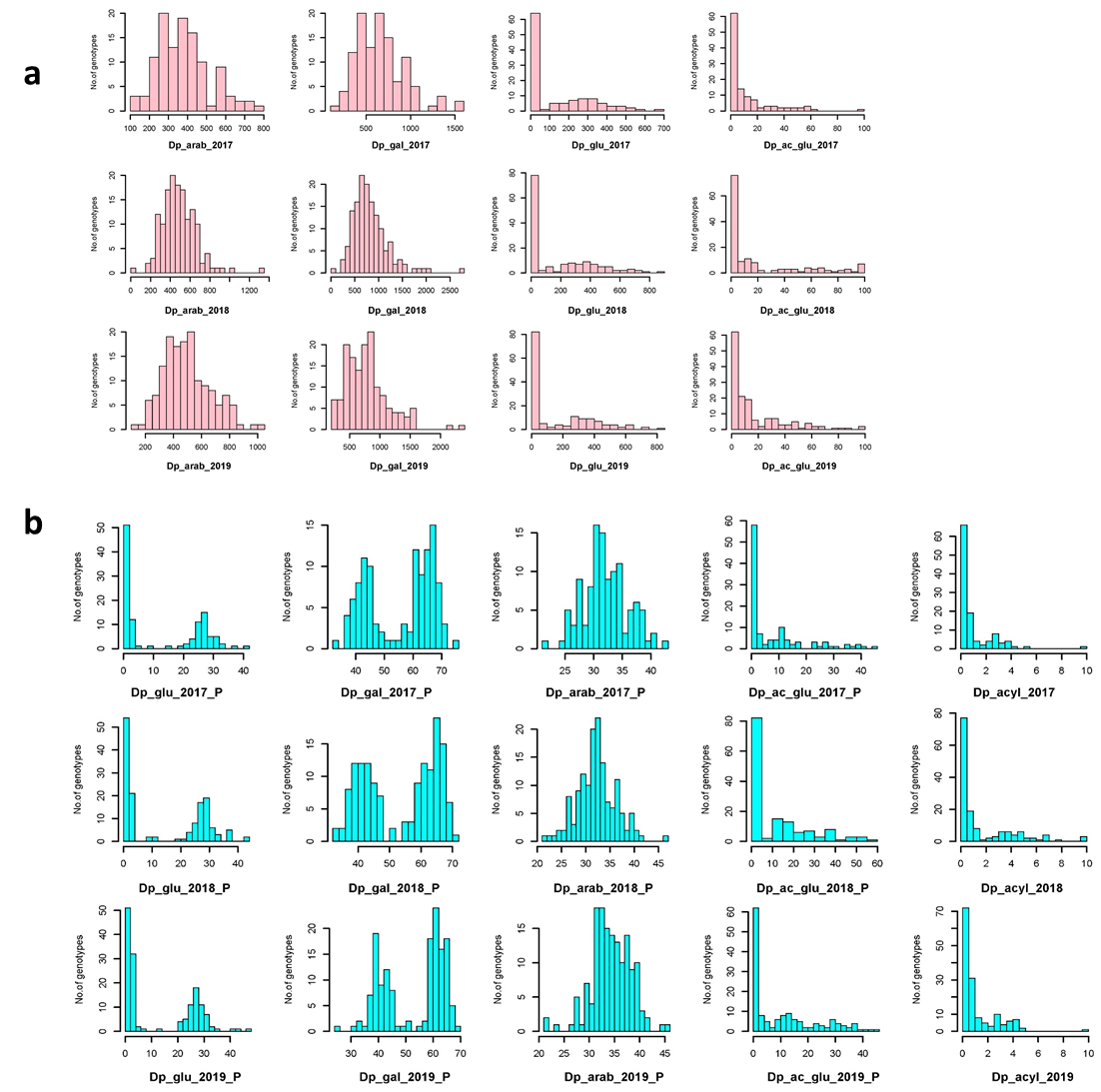


**Figure S3 continue. Phenotypic distribution of concentration** (**ug/g, fresh weight) and relative contribution (%) of delphinidin aglycone based anthocyanins traits over three years. a) concentration; b) relative contribution (%) to the delphinidin group.**


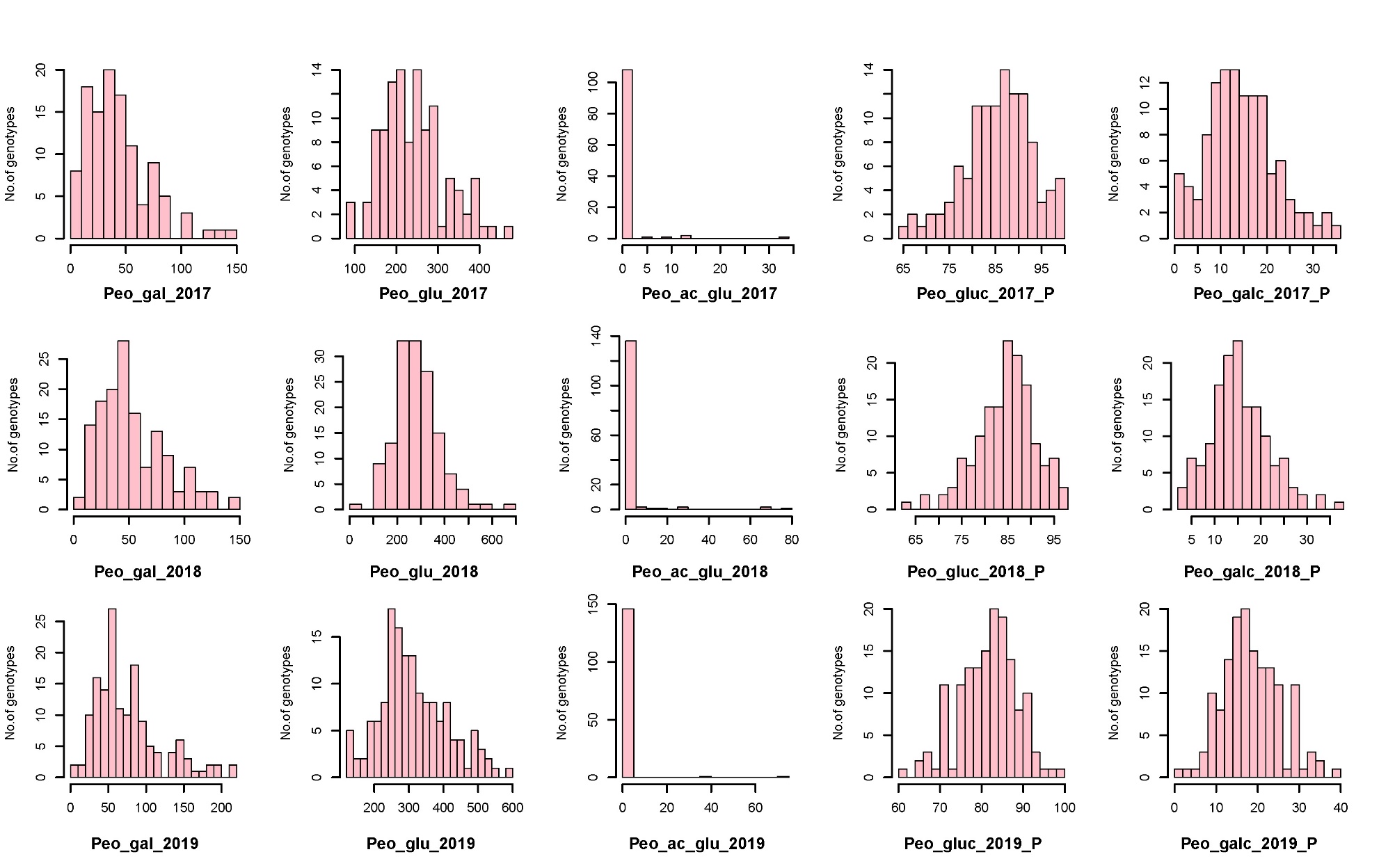


**Figure S3 continue. Phenotypic distribution of concentration** (**ug/g, fresh weight) and relative contribution (%) of peonidin aglycone based anthocyanins traits over three years. a) concentration; b) relative contribution (%) to the peonidin group.**


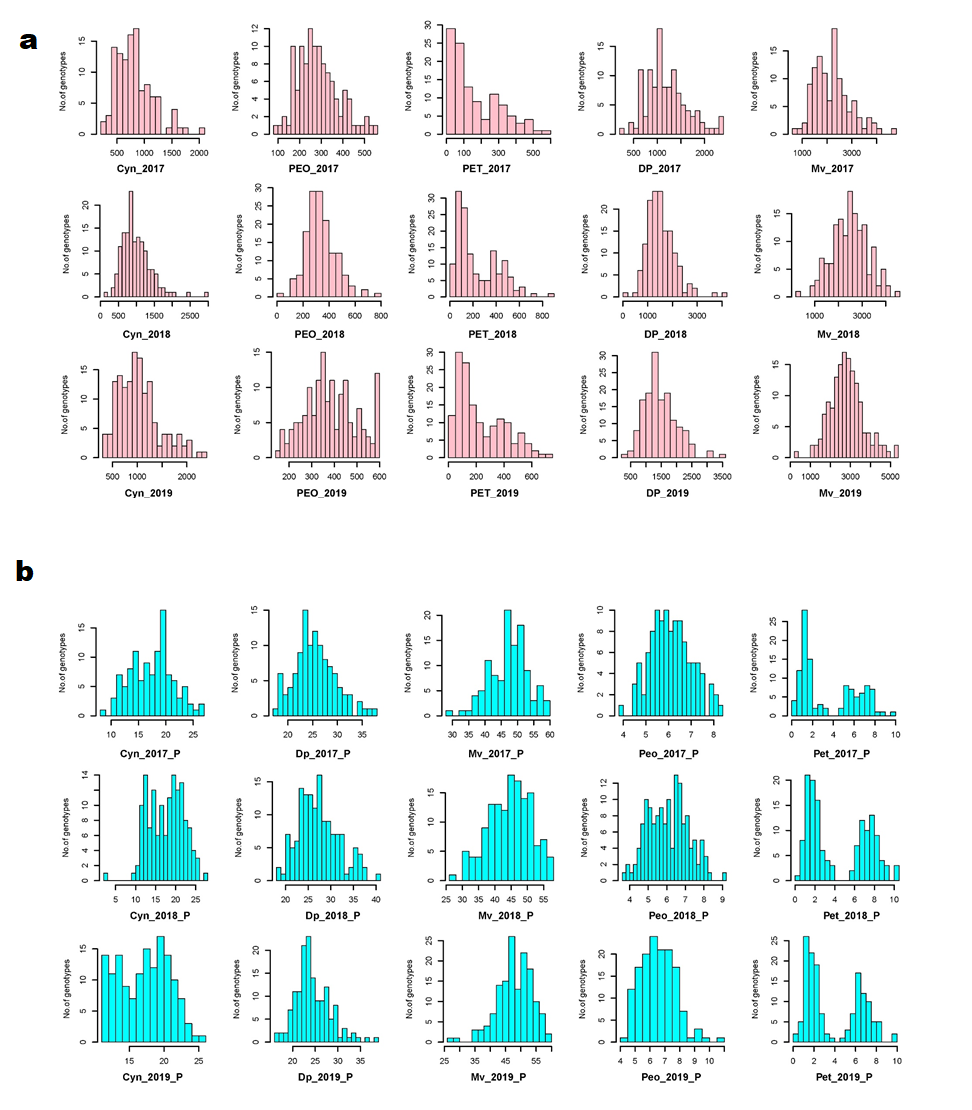


**Figure S4. Phenotypic distribution of concentration** (**ug/g, fresh weight) and relative contribution (%) of anthocyanidin core structure to the totalANC over three years. a) total concentration based on aglycone; b) relative contribution (%) of each aglycone to the total anthocyanin.**


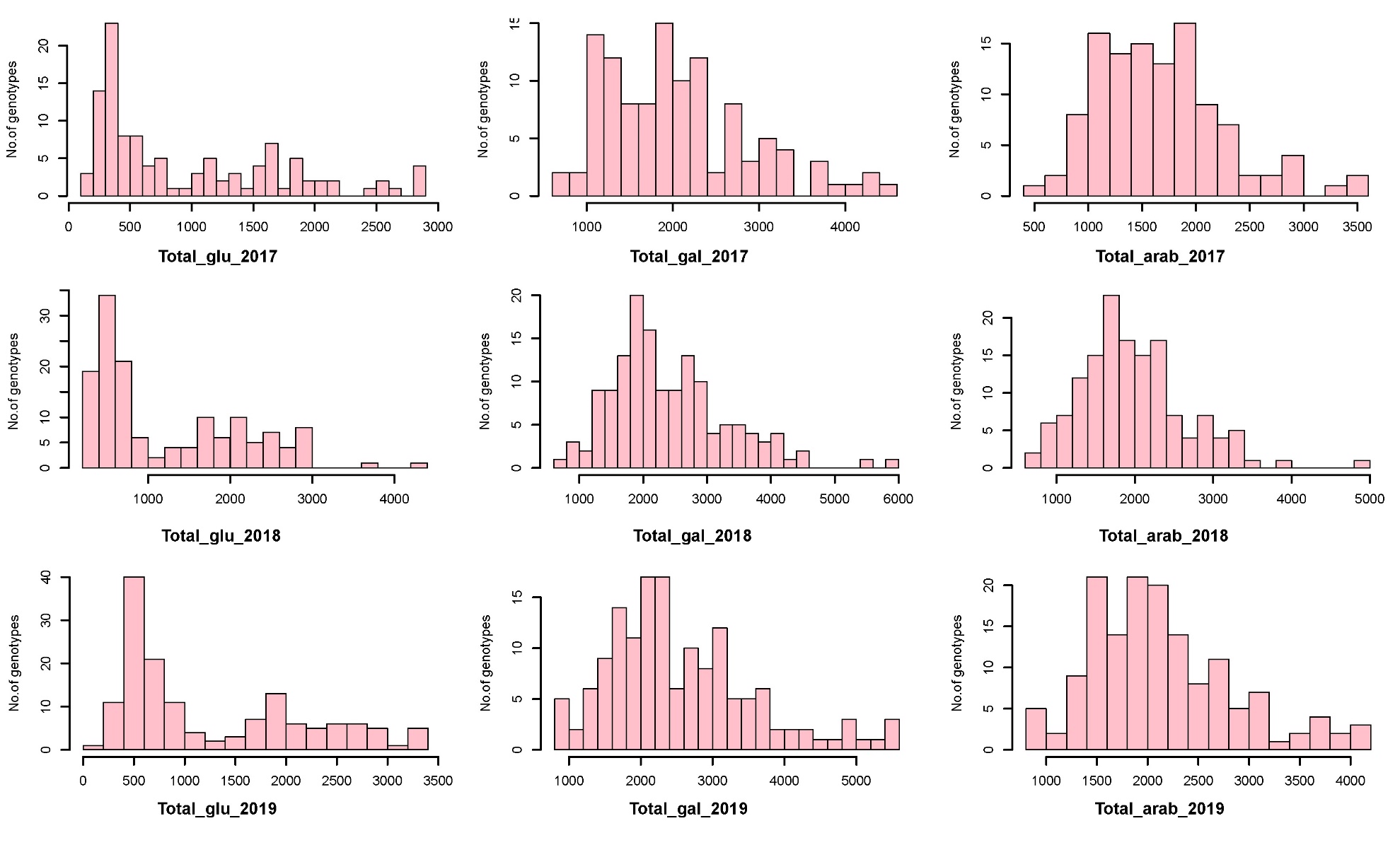


**Figure S5. Phenotypic distribution of total sugar moiety concentration** (**ug/g, fresh weight) over three years.**


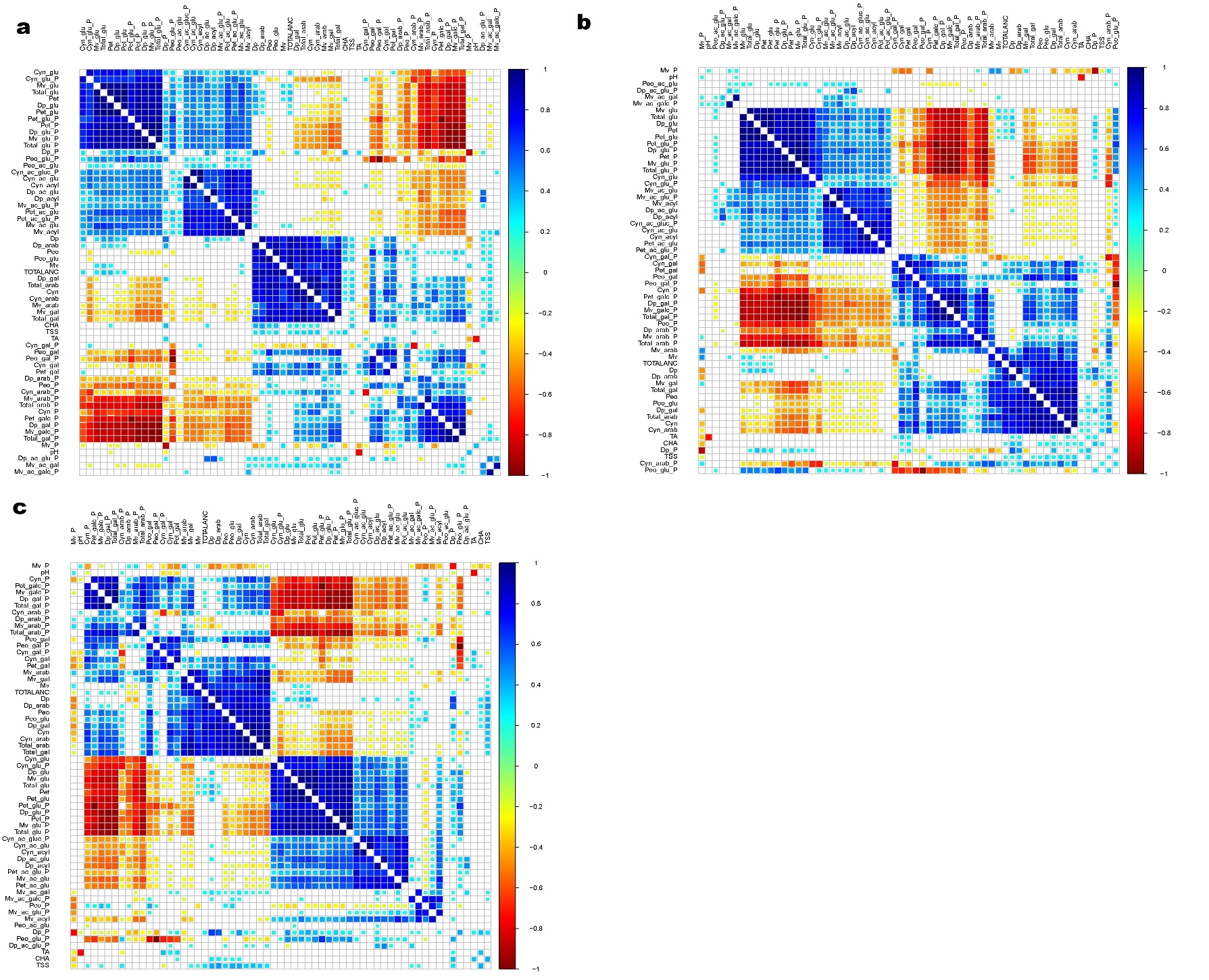


**Figure S6. Correlation between anthocyanins, chlorogenic acid and fruit quality traits over three years. a) correlation between chlorogenic acid, fruit quality and anthocyanins traits in year-2017; b) correlation between chlorogenic acid, fruit quality and anthocyanins traits in year-2018, and c) correlation between chlorogenic acid , fruit quality and anthocyanins traits in year-2019.**


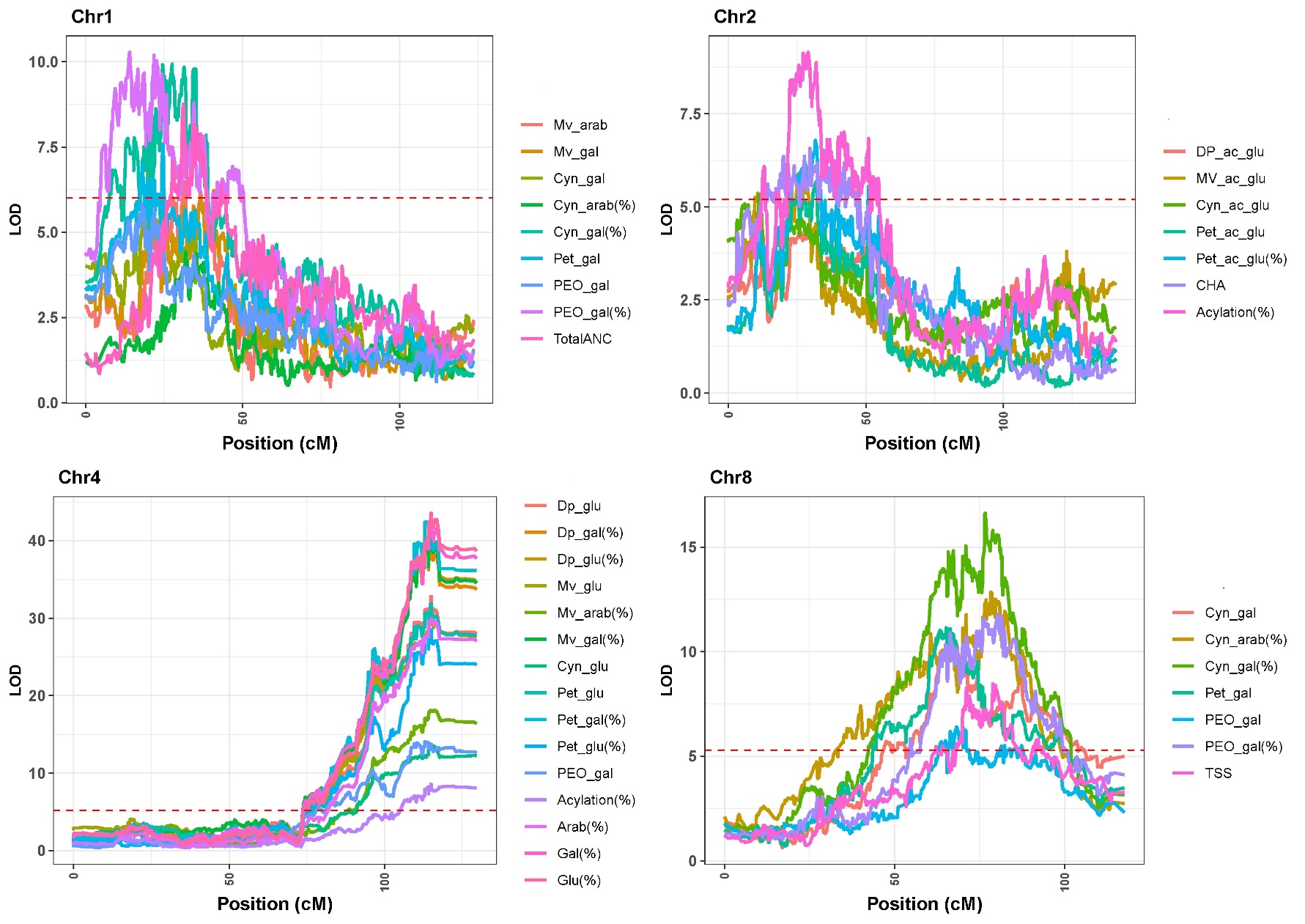


**Figure S7**. Demonstrating overlapping QTLs regions on chromosomes 1, 2, 4 and 8. The red dotted line refers significant threshold level at 1000 permutation and alpha=0.05.


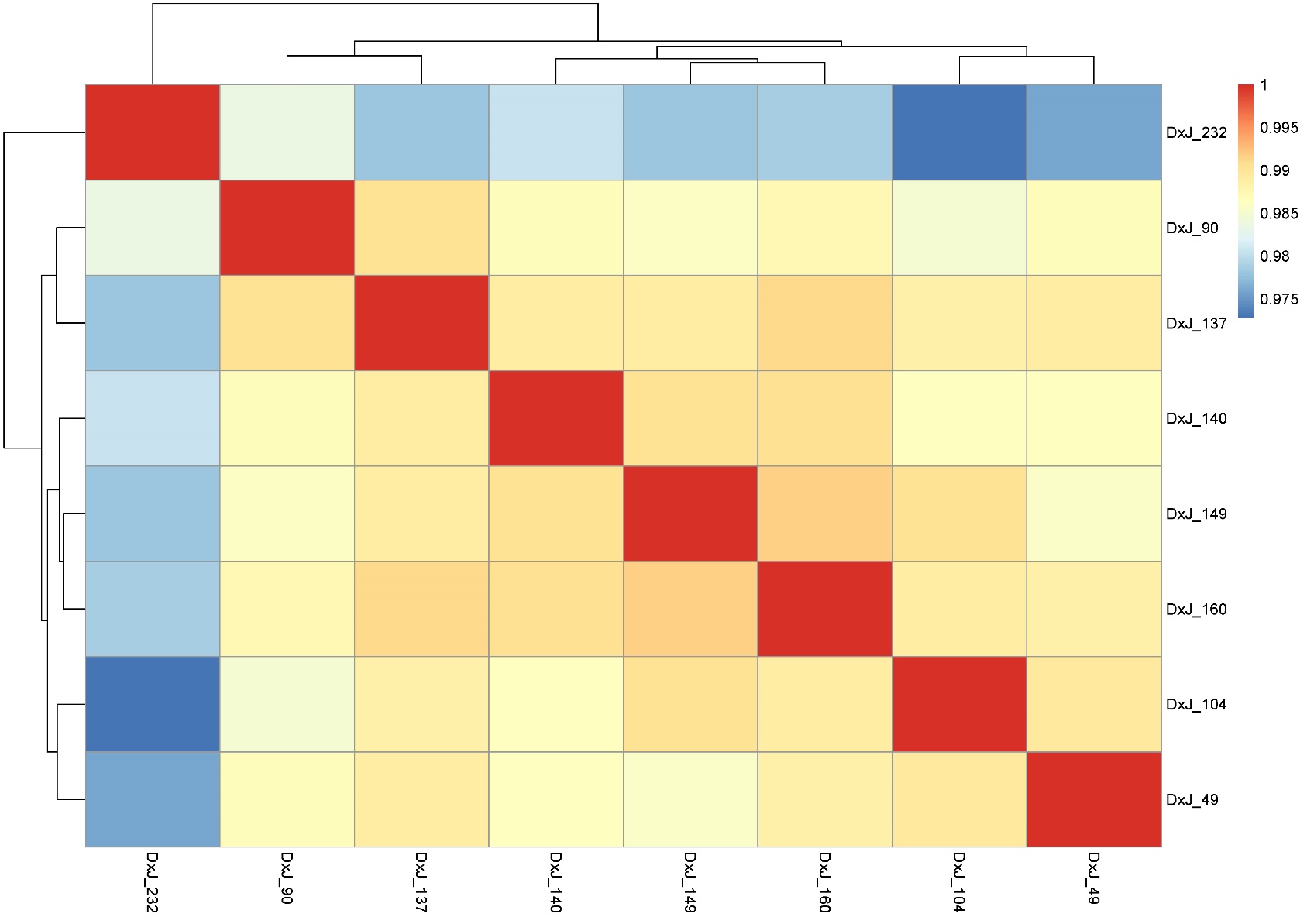


**Figure S8. Hierarchical clustering analysis of RNA-seq data from eight F_1_ genotypes of DSxJ mapping population.**


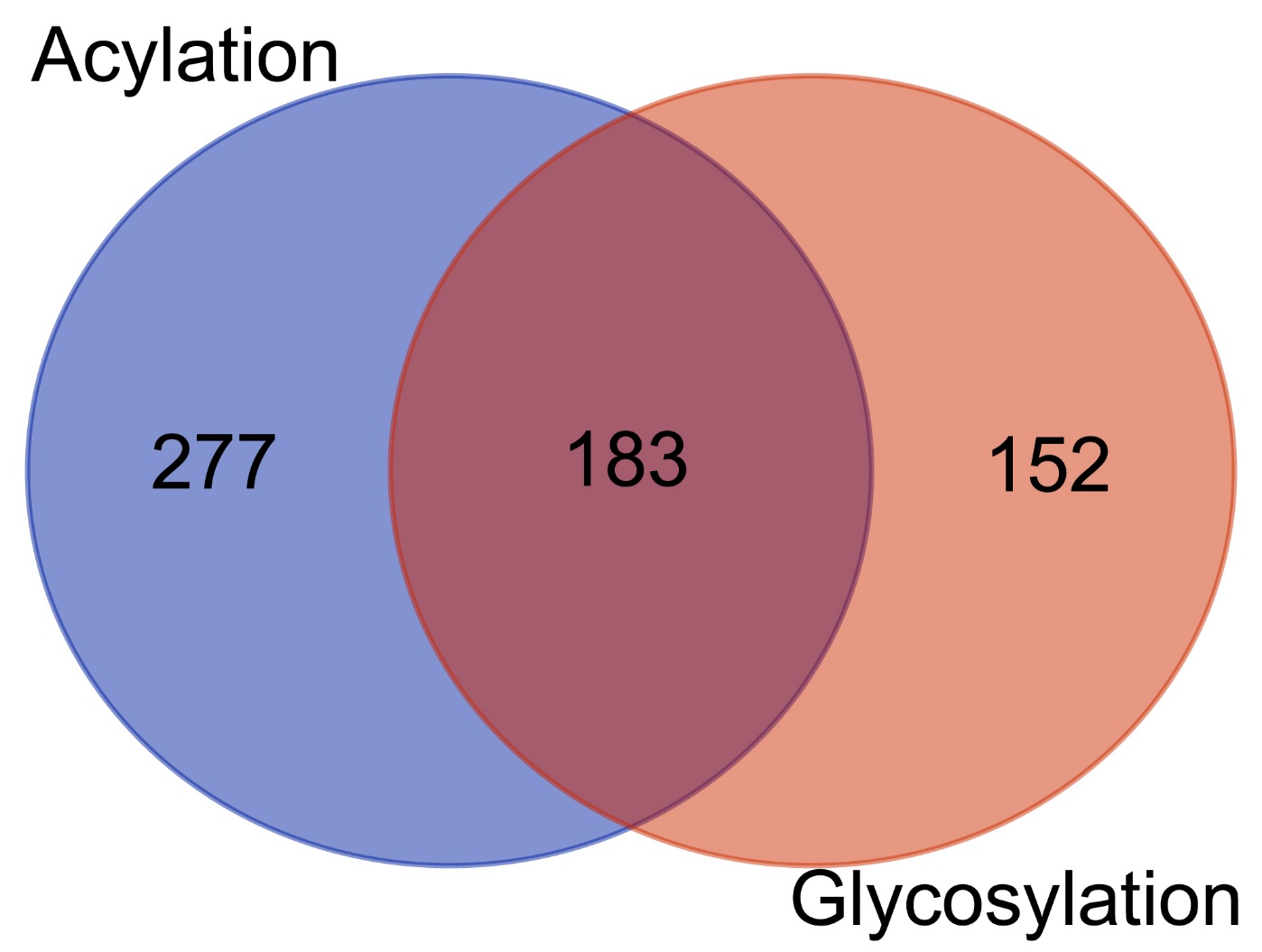


**Figure S9**. Venn diagram showing DEGs identified between samples associated with glycosylation and acylation of anthocyanins. Glycosylation refers the comparison performed between high-glycosylated anthocyanin vs. low-glycosylated anthocyanins group (pink). Acylation refers the comparison performed between acylated-group high-acylated vs. low-acylated group.


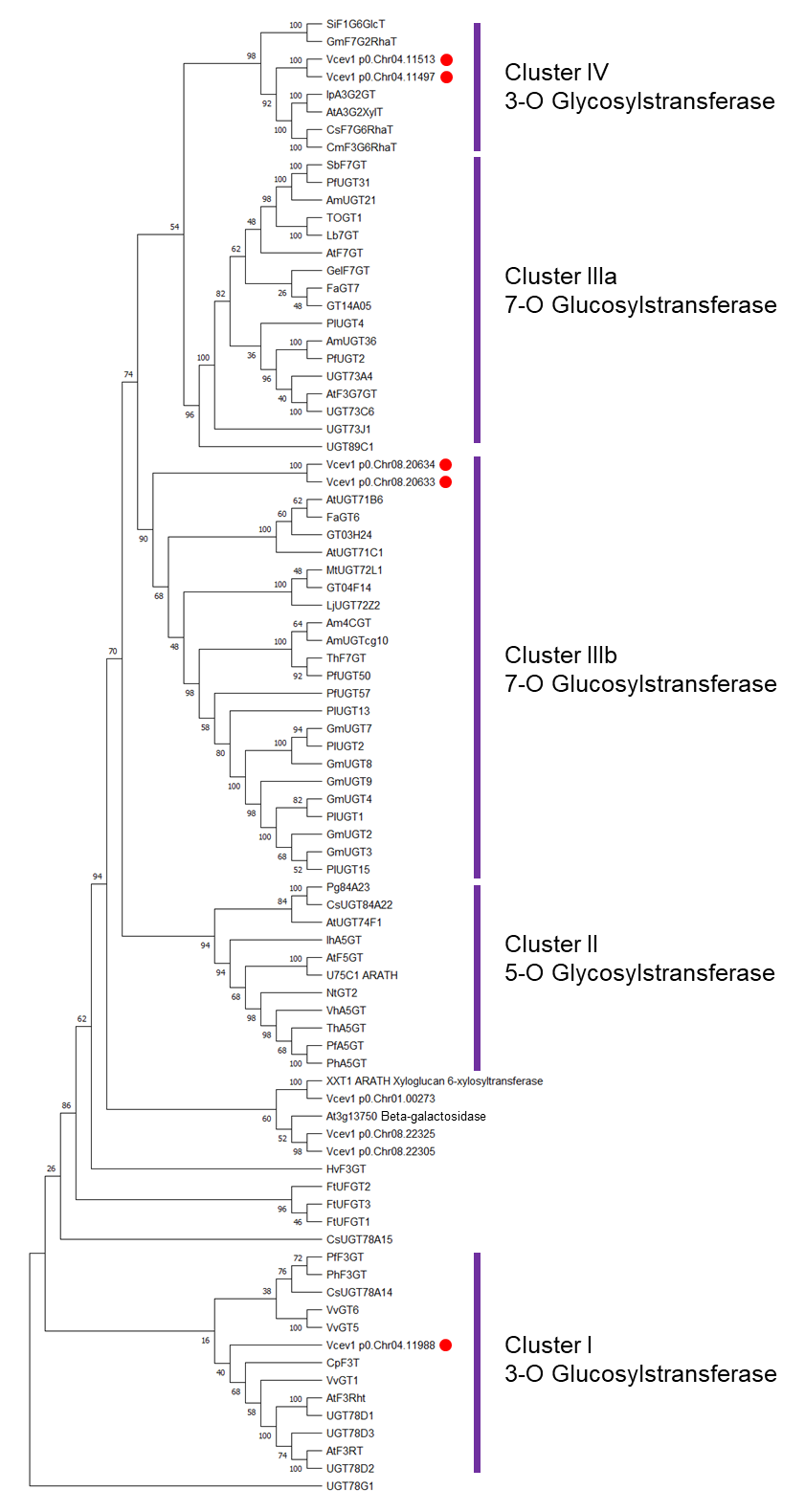


**Figure S10.** Maximum Likelihood phylogenetic tree of selected plant UFGTs and putative blueberry UFGTs identified as DEG within QTL regions. Bootstrap values are percentage from 100 replicates. The Genbank ID for the sequences are reported in **Table S7**. Clades are labeled according to[1] Yao et al., 2019. Red circles indicate blueberry genes clustered with known UFGTs genes.

**
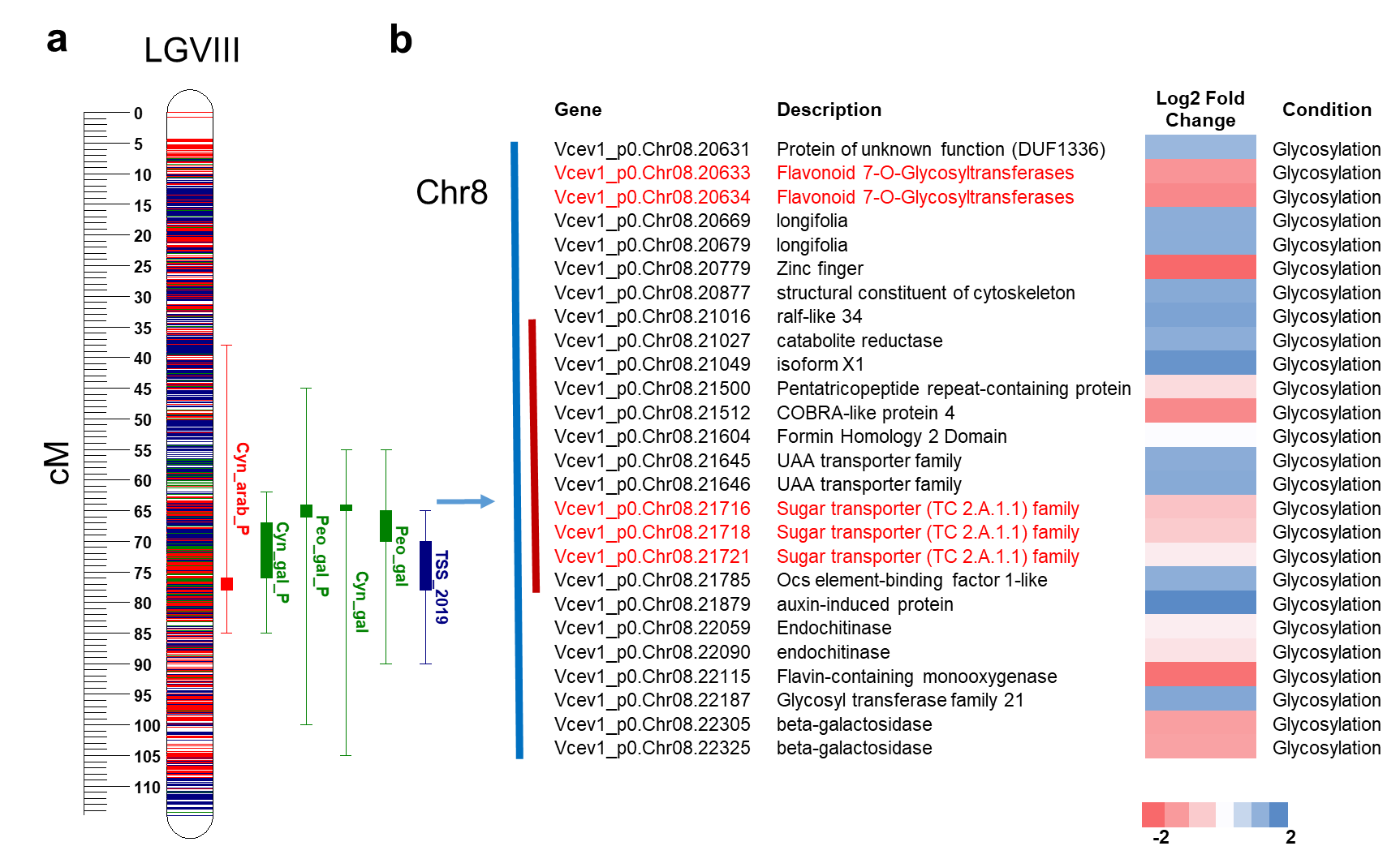
**

**Figure S11.** Integrating QTL mapping with DEGs from RNA-seq analysis. a) Glycosylation related QTLs cluster on chromosome 8. Boxplot represents the QTL position for % cyanidin-arabinoside (red), % cyanidin-galactoside, % peonidin-galactoside and peonidin-galactoside (green) and total soluble solid for year-2019 (blue). The boxplot represents the 95% permutation support interval (the interval where the QTL peak exceeds the LOD threshold established using 1000 permutations and α=0.05). The solid box of the boxplot represents two-LOD support interval. b) List of DEGs in the 95% permutation support interval (blue bar) and two-LOD support interval (red bar). Potential candidate genes are highlighted in red. Log2fold change represents the gene expression levels from down regulated (red) to up-regulated (blue). Condition represents the pairwise comparison performed during gene expression analysis. Glycosylation: high-glycosylated vs. low glycosylated. For each traits, the two-LOD and 95% support interval presented here represent the overlap of the QTL regions detected across three years.

**
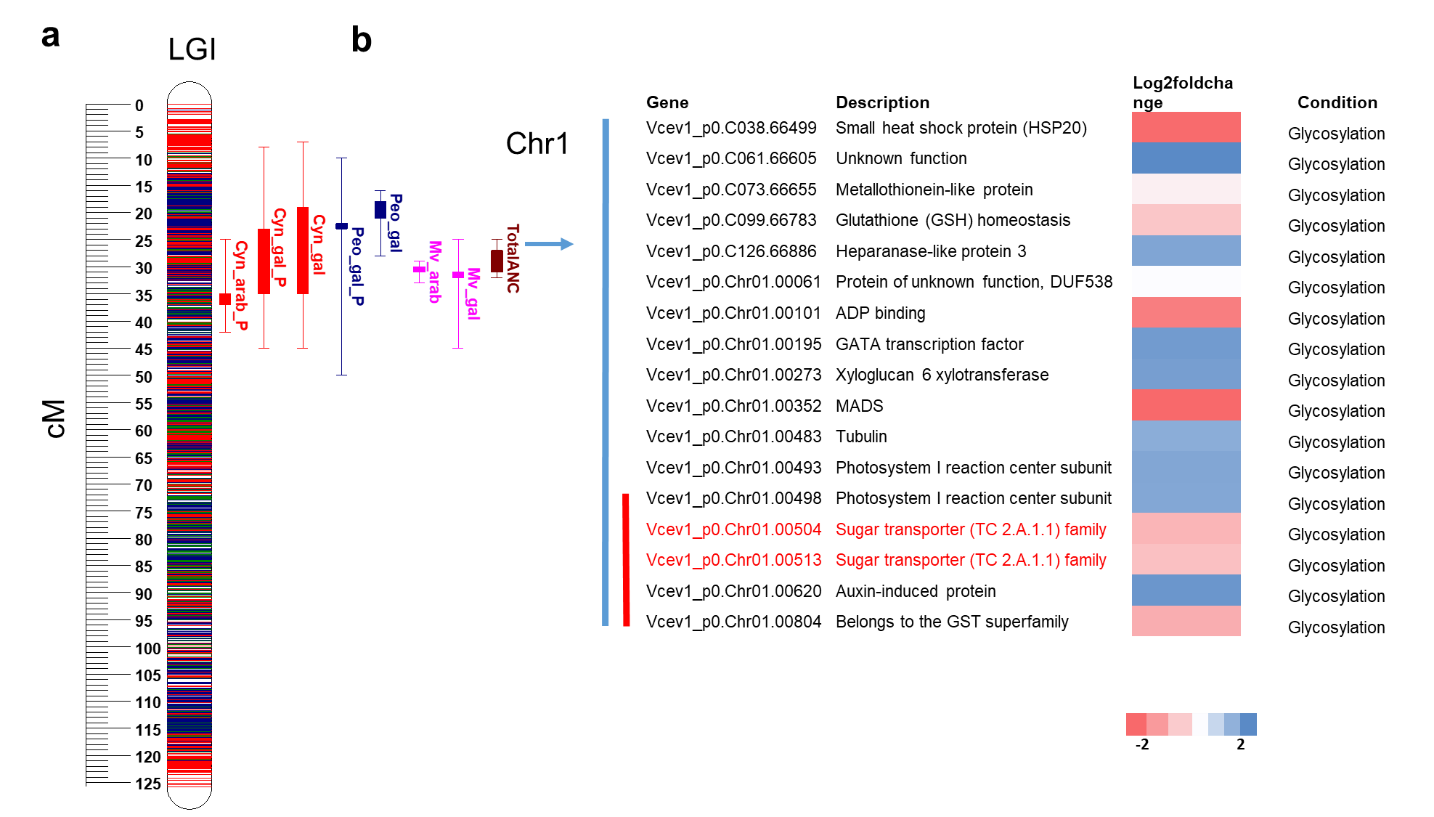
**

**Figure S12.** Integrating QTL mapping with DEGs from RNA-seq analysis. a) Glycosylation related QTLs cluster on chromosome 1. Boxplot represents the QTL position for % cyanidin-arabinoside, % cyanidin-galactoside, cyanidin galactoside (red), % peonidin-galactoside and peonidin-galactoside (blue), malvidin-galactoside and malvidin-arabinoside (violet) and totalANC (brown). The boxplot represents the 95% permutation support interval (the interval where the QTL peak exceeds the LOD threshold established using 1000 permutations and α=0.05). The solid box of the boxplot represents two-LOD support interval. b) List of DEGs in the 95% permutation support interval (blue bar) and two-LOD support interval (red bar). Potential candidate genes are highlighted in red. Log2fold change represents the gene expression levels from down regulated (red) to up-regulated (blue). Condition represents the pairwise comparison performed during gene expression analysis. Glycosylation: high-glycosylated vs. low glycosylated. For each traits, the two-LOD and 95% support interval presented here represent the overlap of the QTL regions detected across three years.


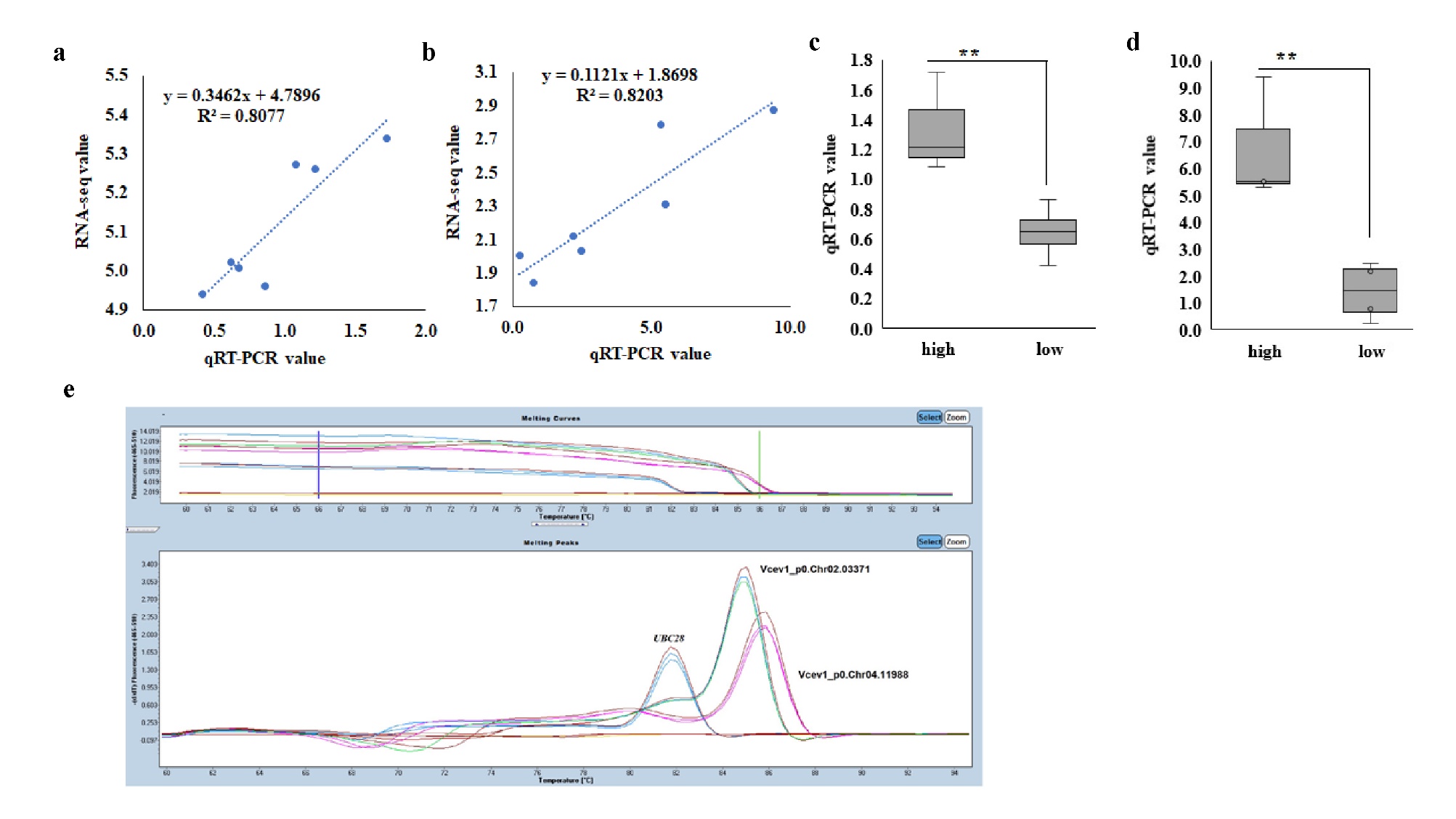


**Figure S13**. Verification of the RNA-seq data expression profile by qRT-PCR. a) Correlation between the qRT-PCR gene expression and RNA-seq value for candidate gene *Vcev1_p0.Chr04.11988;* b) Correlation between the qRT-PCR gene expression and RNA-seq value for candidate gene *Vcev1_p0.Chr02.03371;* c) qRT-PCR expression value for gene *Vcev1_p0.Chr04.11988* in high and low glycosylated samples*;* d) qRT-PCR value for gene *Vcev1_p0.Chr02.03371* in high and low acylated sample*;* e) Melting curve profile of the candidate genes *Vcev1_p0.Chr02.03371, Vcev1_p0.Chr04.11988*, and reference gene UBC28. ** indicated value that were statistically significant at *P < 0.01*. The RNA-seq value represent the log10 (FPKM) value, and the qRT-PCR value represent the 2^^-ΔΔCT^ value.


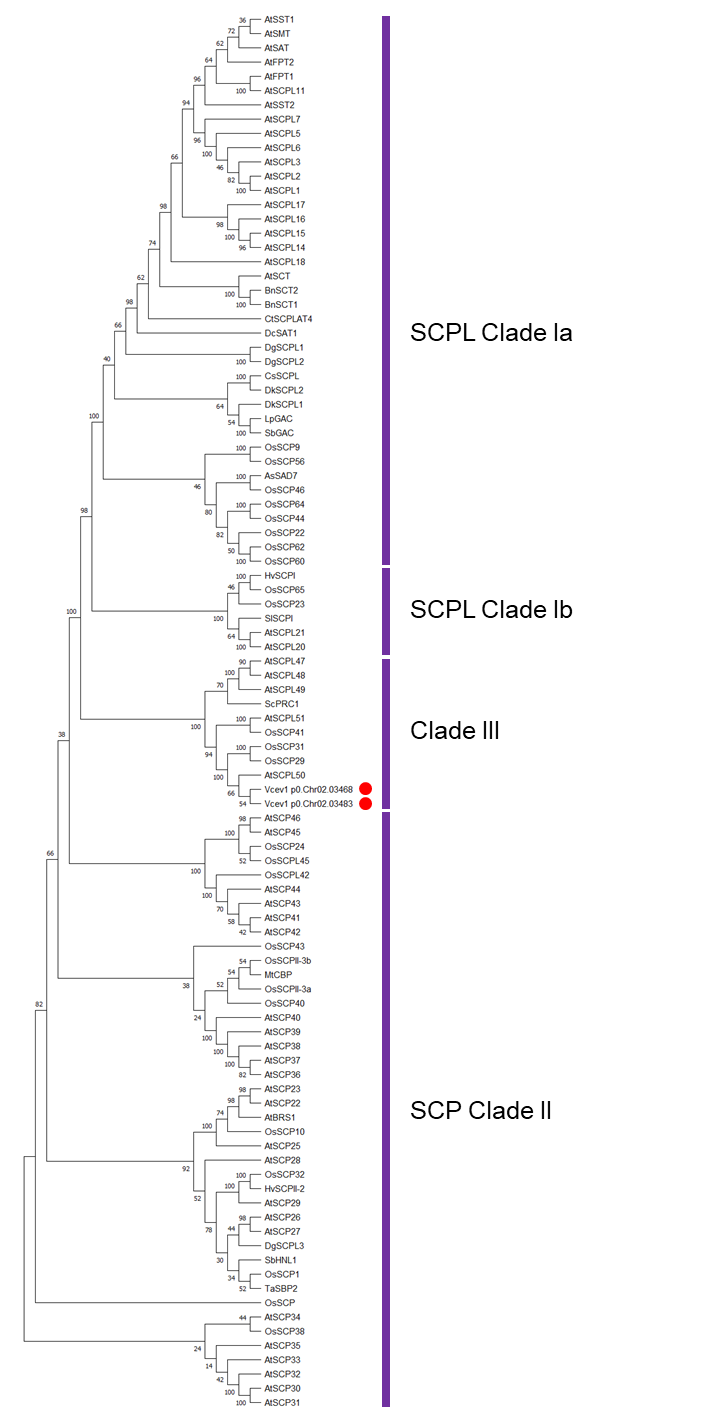


**Figure S14.** Maximum Likelihood phylogenetic tree of selected plant SCPL-SCP genes and putative blueberry SCPL-SCP identified within the chromosome 2 QTL region associated with acylation. Bootstrap values are percentage from 100 replicates. The Genbank ID for the sequences are reported in **Table S8**. Clades are labeled according to [2]. Red circles indicate blueberry genes clustered with known SCP genes.

**References**

1. Yao, P.; Deng, R.; Huang, Y.; Stael, S.; Shi, J.; Shi, G.; Lv, B.; Li, Q.; Dong, Q.; Wu, Q.; et al. Diverse biological effects of glycosyltransferase genes from Tartary buckwheat. *BMC Plant Biology* **2019**, *19*, 1–15.

2. Mugford, S.T.; Qi, X.; Bakht, S.; Hill, L.; Wegel, E.; Hughes, R.K.; Papadopoulou, K.; Melton, R.; Philo, M.; Sainsbury, F.; et al. A Serine Carboxypeptidase-Like Acyltransferase Is Required for Synthesis of Antimicrobial Compounds and Disease Resistance in Oats  . *The Plant Cell* **2009**, *21*, 2473–2484.
